# Convertase-dependent regulation of membrane-tethered and secreted ligands tunes dendrite adhesion

**DOI:** 10.1242/dev.201208

**Published:** 2023-09-18

**Authors:** Nelson J. Ramirez-Suarez, Helen M. Belalcazar, Maisha Rahman, Meera Trivedi, Leo T. H. Tang, Hannes E. Bülow

**Affiliations:** ^1^ Department of Genetics, Albert Einstein College of Medicine, Bronx, NY 10461, USA; ^2^ Dominick P. Purpura Department of Neuroscience, Albert Einstein College of Medicine, Bronx, NY 10461, USA

**Keywords:** Proprotein convertase, Dendrites, *Caenorhabditis elegans*, Menorin, Tethered ligand

## Abstract

During neural development, cellular adhesion is crucial for interactions among and between neurons and surrounding tissues. This function is mediated by conserved cell adhesion molecules, which are tightly regulated to allow for coordinated neuronal outgrowth. Here, we show that the proprotein convertase KPC-1 (homolog of mammalian furin) regulates the Menorin adhesion complex during development of PVD dendritic arbors in *Caenorhabditis elegans.* We found a finely regulated antagonistic balance between PVD-expressed KPC-1 and the epidermally expressed putative cell adhesion molecule MNR-1 (Menorin). Genetically, partial loss of *mnr-1* suppressed partial loss of *kpc-1*, and both loss of *kpc-1* and transgenic overexpression of *mnr-1* resulted in indistinguishable phenotypes in PVD dendrites. This balance regulated cell-surface localization of the DMA-1 leucine-rich transmembrane receptor in PVD neurons. Lastly, *kpc-1* mutants showed increased amounts of MNR-1 and decreased amounts of muscle-derived LECT-2 (Chondromodulin II), which is also part of the Menorin adhesion complex. These observations suggest that KPC-1 in PVD neurons directly or indirectly controls the abundance of proteins of the Menorin adhesion complex from adjacent tissues, thereby providing negative feedback from the dendrite to the instructive cues of surrounding tissues.

## Teaser

**Summary:** The neuronal furin-like proprotein convertase KPC-1 in *Caenorhabditis elegans* requires catalytic activity to non-autonomously regulate the Menorin adhesion complex from adjacent tissues, thereby providing negative feedback during dendrite development.

## INTRODUCTION

Animal behavior relies on functional neuronal networks, which in turn rely on sensation of sensory stimuli and their coordinated processing. Defects in sensory processing of both auditory and mechanosensory modalities have been implicated with neuropsychiatric disease (Antoine et al., 2013; Orefice et al., 2019). The single building block of neural networks are neurons, which receive input through dendrites and pass on information through axonal projections. Crucial functions during neural development are played by cell adhesion molecules that provide adhesive forces to mediate controlled and directed growth of developing neurites. Much is known about the interactions between cell adhesion molecules of adjacent cells as well as their adhesion-independent functions (Moreland and Poulain, 2022), but how adhesive interactions are negatively regulated is a much less understood, yet equally important aspect to guarantee coordinated neuronal development.

**Fig. 1.**
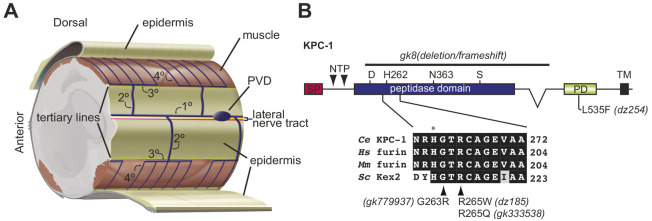
**A genetic modifier screen for genes that function in concert with the KPC-1 proprotein convertase during somatosensory dendrite development.** (A) Schematic of PVD somatosensory neurons in their normal tissue context. The tertiary lines denote the boundaries between the lateral epidermis and the muscle quadrants, and the lateral nerve tract comprising the processes of the ALA and CAN cells is indicated. Modified after Salzberg et al. (2013). (B) Schematic of KPC-1 (note that KPC-1 refers to the shorter isoform KPC-1A described by Salzberg et al., 2014) with relevant amino acids of the catalytic triad and used alleles indicated (asterisk: H262). Partial alignment of KPC-1 (NP_001021101), human furin (NP_002560.1), mouse furin (NP_001074923.1) and yeast Kex2 (NP_014161) is shown with the amino acid number on the right. The asterisk indicates H262, which is part of the catalytic triad. SP, signal peptide; PD, P domain; TM, transmembrane domain.

Conserved mechanisms coordinate dendrite formation in neurons of both vertebrates and invertebrates (Dong et al., 2015; Lefebvre et al., 2015; Lefebvre, 2021). For example, the pair of multidendritic PVD somatosensory neurons in the nematode *Caenorhabditis elegans* are a well-established model for studying dendrite development (reviewed by Inberg et al., 2019; Sundararajan et al., 2019). These neurons each cover one side of the body surface of the nematode, with the exception of the head and neck region, which is covered by a structurally related somatosensory neuron called FLP. In addition to a single axon that both neurons send into the right ventral nerve cord, a stereotypic dendritic tree develops with primary, secondary, tertiary and quaternary dendrites branching at characteristic right angles (Fig. 1A) (Oren-Suissa et al., 2010; Smith et al., 2010; Albeg et al., 2011). After initial outgrowth of the primary dendrite along the lateral nerve tract, orthogonal secondary dendrites branch off in a ventral and dorsal direction. When the secondary branches reach the sublateral line where the lateral epidermis abuts the muscle quadrants, tertiary branches form orthogonally to the secondary branch and in parallel to the primary branch. Eventually, orthogonal quaternary branches emanate, sandwiched between the epidermis and the muscle cells towards the dorsal and ventral midlines without ever crossing them (Fig. 1A). Owing to their similarity to candelabras, the individual units of the dendritic trees have been termed menorahs (Oren-Suissa et al., 2010). The formation of these menorah-like dendritic trees requires the Menorin adhesion complex, which is formed by the skin-derived cell adhesion molecules SAX-7 (also known as L1CAM) and MNR-1 (or Menorin) (Dong et al., 2013; Salzberg et al., 2013). SAX-7 is produced by the epidermis and localized along the sublateral line as well as in a striped pattern in the epidermis along the sarcomers of the muscle, essentially prepatterning the path for growing tertiary and quaternary dendrites (Dong et al., 2013; Salzberg et al., 2013; Liang et al., 2015). Together with the muscle-derived, diffusible LECT-2 (also known as Chondromodulin II) chemokine (Diaz-Balzac et al., 2016; Zou et al., 2016), these three factors are believed to form an adhesion complex with the DMA-1 leucine-rich transmembrane receptor on PVD neurons to establish the elaborate dendritic trees (Fig. 1A) (reviewed by Inberg et al., 2019; Sundararajan et al., 2019).

Proprotein convertases are a class of proteases that cleave proteins at characteristic dibasic motifs (reviewed by Artenstein and Opal, 2011; Seidah, 2011; Seidah and Prat, 2012). They are synthesized as proproteins and undergo autocatalytic cleavage and activation in appropriate cellular compartments. For example, the furin proprotein convertase has been shown to process insulin-like peptides in mammals (Smeekens et al., 1992). The closest homolog to furin in *C. elegans* is the proprotein convertase KPC-1 (Thacker and Rose, 2000), which also has been shown to process insulin-like peptides in nematodes (Hung et al., 2014), suggesting a conservation of function. Furin has been implicated in dendrite development of mice, in which it is believed to facilitate the cell-autonomous maturation of pro-BDNF to BDNF (Zhu et al., 2017). In *C. elegans*, KPC-1 affects dendrite development of multidendritic neurons, including that of PVD (Schroeder et al., 2013), and functions genetically as a negative regulator of the Menorin pathway (Salzberg et al., 2014). Subsequent studies suggested that KPC-1 antagonizes the function of the Menorin complex by decreasing DMA-1 receptor levels on the PVD membrane of *kpc-1* mutant animals (Dong et al., 2016). The observed overabundance of the DMA-1 receptor on the PVD membrane in *kpc-1* mutants was suggested to be the result of defects in KPC-1-dependent endocytosis (Dong et al., 2016). The endocytic function of KPC-1 was shown to require self-activation through autocatalytic processing (Dong et al., 2016). However, it remained unresolved (1) whether proteolytic activity of KPC-1 is required beyond autocatalytic activation and (2) how KPC-1 regulates DMA-1 function or localization.

Using a combination of genetic and biochemical approaches, we discovered that the *kpc-1*-dependent recruitment/retention of DMA-1 on the PVD membrane is dependent on the putative cell adhesion molecule MNR-1. Intriguingly, KPC-1 from PVD dendrites negatively regulates MNR-1 function in the epidermis, which most likely dissociates the Menorin ligand-receptor complex. We propose that the resulting reduced adhesiveness of PVD dendrites and recycling of the DMA-1 receptor facilitates neurite outgrowth. Our findings provide the first instance of a proprotein convertase functioning across tissues to regulate cell-cell interactions during neural development.

## RESULTS

### Reducing *mnr-1* function suppresses partial loss of *kpc-1* function

**Fig. 2.**
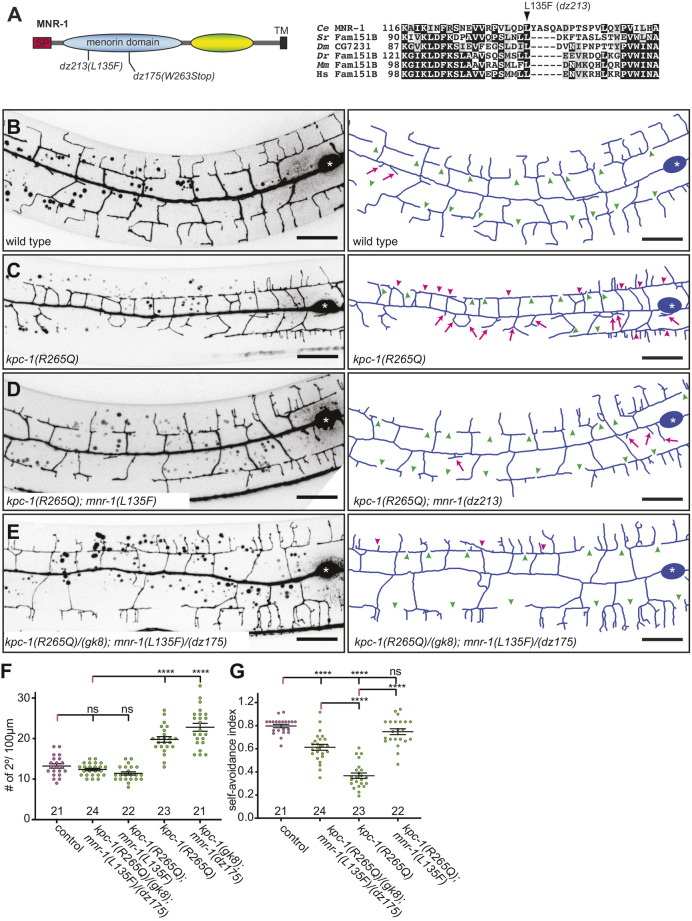
**Reducing MNR-1 function alleviates partial *kpc-1* loss-of-function defects.** (A) Schematic of MNR-1 with the used alleles indicated. SP, signal peptide; TM, putative transmembrane domain. Partial alignment of the Menorin (formerly DUF2181) domain showing the environs of L135 mutated in the *dz213* missense allele. Amino acid numbers are indicated and the accession numbers are: *C. elegans* (*Ce*), NP_507990.1; *Salpingoeca rosetta* (*Sr*), F2TZf0; *Drosophila melanogaster* (*Dm*), NP_995645; *Danio rerio* (*Dr*), CAM14040; *Mus musculus* (*Mm*), NP_001157099; *Homo sapiens* (*Hs*), AAQ88623. (B-E) Fluorescence micrographs with schematics (right) of the indicated genotypes. PVD is visualized by the *wdIs52* transgene. The cell body is marked by asterisks. Green arrowheads indicate gaps and pink arrowheads indicate defects in self-avoidance between neighboring 3° dendrites. Pink arrows show short, immature 2° dendrites. Scale bars: 20 µm. (F,G) Quantification of the number of 2° dendrites in a 100 µm segment anterior to the cell body and self-avoidance defects (expressed as self-avoidance index) in the genotypes indicated. The numbers of animals analyzed are indicated. For F: control (*n*=21), *kpc-1(R265Q)/(gk8);mnr-1(L135F)/(dz175)* (*n*=24), *kpc-1(R265Q);mnr-1(L135F)* (*n*=22), *kpc-1(R265Q)* (*n*=23) and *kpc-1(gk8);mnr-1(dz175)* (*n*=21). For G: control (*n*=21), *kpc-1(R265Q)/(gk8);mnr-1(L135F)/(dz175)* (*n*=24), *kpc-1(R265Q)* (*n*=23) and *kpc-1(R265Q);mnr-1(L135F)* (*n*=22). Error bars indicate the s.e.m. and statistical significance was calculated using one-way ANOVA with Šidák's correction for multiple comparisons. ns, not significant; ****P≤0.0001.

Complete loss of KPC-1 function causes defects in dendrite branch extension, branch number and self-avoidance of tertiary dendrites in PVD neurons, likely owing to increased adhesion of the Menorin complex (Schroeder et al., 2013; Salzberg et al., 2014; Dong et al., 2016). To identify additional genes that function with *kpc-1* during dendrite morphogenesis, we performed a genetic modifier screen of a *kpc-1* partial-loss-of-function allele (*gk333538*, hereafter named R265Q). This allele changes a conserved arginine residue in close proximity to the catalytic histidine 262, and results in a significant increase in the number of secondary branches and defects in self-avoidance of tertiary branches (Fig. 1B, Fig. 2) (Salzberg et al., 2014). Of ten enhancer and suppressor mutants isolated in this screen, one allele suppressed, whereas nine alleles enhanced the *kpc-1* loss-of-function phenotype (Table 1). We also identified 16 modifier mutations, which showed distinct phenotypes and have been (Tang et al., 2019; Tang et al. 2021) or will be described in the future. Of the enhancer mutations, eight alleles were either intragenic enhancers of *kpc-1* or closely linked, and resulted in *kpc-1*-null-mutant-like phenotypes in PVD dendrites in combination with the *kpc-1(R265Q)* partial loss-of-function allele, suggesting that they likely affected the *kpc-1* locus (Table 1). The remaining two alleles likely represented extragenic modifiers (one enhancer and one suppressor) of *kpc-1(R265Q)*. Here, we focus on the *dz213* allele, which completely suppresses all *kpc-1(R265Q)* mutant phenotypes.

**Table 1.**
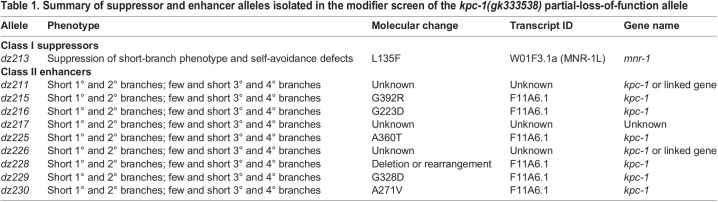
Summary of suppressor and enhancer alleles isolated in the modifier screen of the *kpc-1(gk333538)* partial-loss-of-function allele

Using whole-genome sequencing, mapping and transformation rescue, we identified the molecular lesion of the *dz213* suppressor allele as a missense mutation in *mnr-1* (Table 1, Fig. 2A; Fig. S1A,B, Fig. S2A,B). This missense mutation changes leucine 135 to a phenylalanine, a residue within the Menorin domain (previously DUF2181), which is conserved from choanoflagellates to humans (Fig. 2A). The L135F change in MNR-1 suppressed both the branching and self-avoidance defects of PVD in *kpc-1(R265Q)* mutant animals (Fig. 2B-D,F). Several observations indicate that *mnr-1(dz213)* [hereafter referred to as *mnr-1(L135F*)] is a partial loss of function allele. First, *mnr-1(L135F)* failed to complement the putative null allele *mnr-1(dz175)*, both with regard to the number of branches and the dendrite self-avoidance defects (Fig. 2E-G). Second, two copies of the mutant allele (*L135F*) were required for suppression of the *kpc-1(R265Q)* PVD phenotype, showing that *mnr-1(L135F)* is recessive (Fig. 2D,E). Third, transgenic expression of a wild-type *mnr-1* cDNA in the epidermis rescued the *mnr-1(L135F)* mutant, i.e. it reversed the suppression of the *kpc-1(R265Q); mnr-1(L135F)* double mutant, and overexpression of a *mnr-1(L135F)* mutant cDNA partially rescued *mnr-1(dz175)* null mutant animals (Fig. S2A,B). Some MNR-1 function is required for efficient suppression of partial loss of *kpc-1* function because (1) the suppression is dosage sensitive (Fig. 2G); (2) the *mnr-1(dz175)* putative null allele could not suppress the *kpc-1(R265Q)* hypomorphic phenotype (Fig. S2C); and (3) overexpression of the *mnr-1(L135F)* mutant cDNA driven by an epidermal promoter completely rescued the *mnr-1* null phenotype in *kpc-1(R265Q); mnr-1(dz175)* double-mutant animals (data not shown). Finally, the *mnr-1(L135F)* allele is temperature sensitive because *mnr-1(L135F)* mutant animals displayed PVD phenotypes at the non-permissive temperature of 25°C that were indistinguishable from the *mnr-1* null phenotype, but no phenotype at all at the permissive temperature of 20°C (Fig. S2D). All suppression conferred by the *mnr-1(L135F)* allele in *kpc-1(R265Q); mnr-1(L135F)* double mutants was lost at the non-permissive temperature compared with at the permissive temperature (Fig. S2D), also supporting the notion that (1) *mnr-1(L135F)* is dosage sensitive and (2) that some MNR-1 function is required to suppress a partial loss of KPC-1 function. Thus, we conclude that *mnr-1(L135F)* is a recessive, partial-loss-of-function allele of *mnr-1*, which can suppress the defects of a partial-loss-of-function allele of *kpc-1*. Importantly, only reducing but not eliminating *mnr-1* function can compensate for reduced *kpc-1* function.

We next asked whether, conversely, suppression of the *kpc-1* partial-loss-of-function phenotype in the *mnr-1(L135F)* allele is dependent on some *kpc-1* function. Existing *kpc-1* alleles form an allelic series as follows: *gk8*=*gk779937*>*dz185*>*gk333538*>*dz254*, where *gk8* represents the putative null phenotype with the strongest phenotype and *dz254* represents the weakest phenotype (Salzberg et al., 2014; Rahman et al., 2022) (Fig. S2C,E-G). The strong-loss-of-function-alleles display entrapment of the dendrites in the lateral epidermis, whereas hypomorphic alleles display primarily self-avoidance defects (Salzberg et al., 2014; Dong et al., 2016). We found that the *mnr-1(L135F)* allele completely suppressed the weaker *kpc-1* alleles (*dz254* and *gk333538*), but only partially suppressed the intermediate *kpc-1(dz185)* allele (Fig. S2C). The putative *kpc-1(gk8)* null allele (catalytic domain deletion) and the *kpc-1(gk779937)* (G263R) strong-loss-of-function allele were not suppressed by the *mnr-1(L135F)* allele (Fig. S2C). In conclusion, the suppression of *kpc-1* by loss of MNR-1 function is (1) sensitive to both the dosage of MNR-1 and KPC-1, and (2) requires residual function of both genes.

### Increased *mnr-1* function mimics *kpc-1* loss-of-function phenotypes

**Fig. 3.**
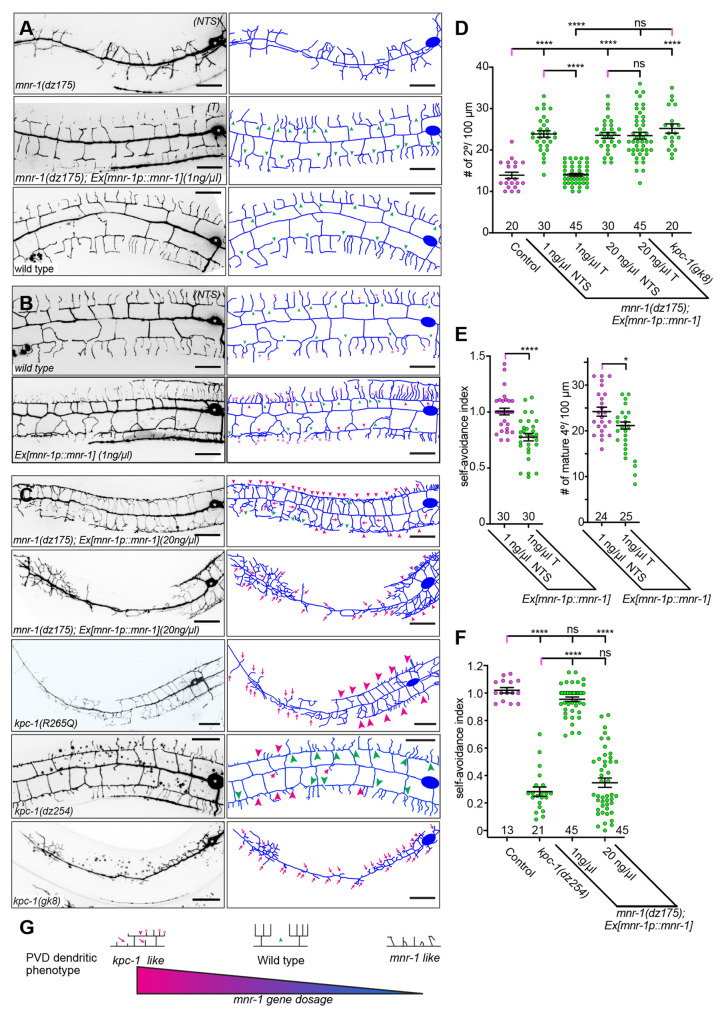
**An antagonistic balance between MNR-1 and KPC-1 shapes dendrites.** (A-C) Fluorescence micrographs with schematics (right) of the indicated genotypes. PVD is visualized by the *wdIs52* transgene. The cell body is marked by asterisks. Note that in C, the *mnr-1-*overexpressing strains display a range of phenotypes from severe self-avoidance defects to complete entrapment of dendrites along the primary dendrite. Similarly, partial-loss-of-function alleles of *kpc-1* show a range of self-avoidance defects, whereas the *kpc-1(gk8)* null allele shows the entrapment phenotype. Green arrowheads indicate gaps and pink arrowheads indicate defects in self-avoidance between neighboring 3° dendrites. Pink arrows and pink open circles show short, immature 2° and 4° dendrites, respectively. Scale bars: 20 µm. NTS, non-transgenic siblings; T, transgenic siblings. (D-F) Quantification of the number of 2° dendrites in a 100 µm segment anterior to the cell body or the self-avoidance index in the genotypes indicated. The number of animals analyzed is indicated. For D: control (*n*=20), 1 ng/µl *Ex[mnr-1p::mnr-1]* NTS (*n*=30), 1 ng/µl *Ex[mnr-1p::mnr-1]* T (*n*=45), 20 ng/µl *Ex[mnr-1p::mnr-1]* NTS (*n*=30), 20 ng/µl *Ex[mnr-1p::mnr-1]* T (*n*=45), *kpc-1(gk8)* (*n*=20). For E: 1 ng/µl *Ex[mnr-1p::mnr-1]* NTS [*n*=30 (left) and 24 (right)], 1 ng/µl *Ex[mnr-1p::mnr-1]* T [*n*=30 (left) and 25 (right)]. For F: control (*n*=13), *kpc-1(dz254)* (*n*=21), 1 ng/µl *Ex[mnr-1p::mnr-1]* (*n*=45), 20 ng/µl *Ex[mnr-1p::mnr-1]* (*n*=45). Error bars indicate the s.e.m. and statistical significance was calculated using one-way ANOVA with Šidák's correction for multiple comparisons (D,F) or unpaired, two-tailed *t*-tests (E). ns, not significant; *P≤0.05; ****P≤0.0001. (G) Schematic illustrating how *mnr-1* dosage regulates dendrite morphogenesis. The green arrowhead indicates gaps and the pink arrowhead indicates defects in self-avoidance between neighboring 3° dendrites. Pink arrows and pink open circles show short, immature 2° and 4° dendrites, respectively.

How could loss of MNR-1 function alleviate defects due to reduced KPC-1 function? It has previously been suggested that KPC-1 can negatively regulate extracellular adhesion of dendritic growth cones to the growth substrate (the epidermis) by promoting endocytosis of the DMA-1 receptor (Dong et al., 2016). Our genetic results raised the possibility that increasing the expression of *mnr-1* could have similar effects: adhesive forces exerted by overactivation of the Menorin complex from the epidermis could prevent dendrite extension. To test this hypothesis, we first expressed a fosmid containing *mnr-1* under control of most, if not all, regulatory regions at low doses (1 ng/µl) in *mnr-1(dz175)* null mutants and found that it, as expected, completely rescued the PVD defects of *mnr-1* mutant animals (Fig. 3A,D). Interestingly, the same *mnr-1-*expressing transgene in a wild-type background resulted in self-avoidance defects and a reduced number of fully extended 4° branches, reminiscent of *kpc-1* partial-loss-of-function alleles (Fig. 3B,E). Moreover, transgenic overexpression of *mnr-1* at high doses (20 ng/µl) resulted in a dramatic transformation of the PVD dendritic tree from a *mnr-1-*like phenotype to a *kpc-1*-like mutant phenotype (Fig. 3C). Specifically, we observed a strong increase in the number of short, immature secondary branches and self-avoidance defects that were indistinguishable from *kpc-1* null mutants (Fig. 3C,D,F). Note that when *kpc-1* function was completely lost [*kpc-1(gk8)*], dendrites became essentially trapped in the vicinity of the primary dendrite and self-avoidance defects could not be detected (Fig. 3C). Similar results were obtained when *mnr-1* was expressed under control of an epidermis-specific promoter (Fig. 3C). Together with the loss-of-function studies, these results suggest a tightly regulated antagonistic balance between the functions of MNR-1 and KPC-1 (Fig. 3G).

### MNR-1 is necessary and sufficient for cell-surface localization of DMA-1 in PVD dendrites

**Fig. 4.**
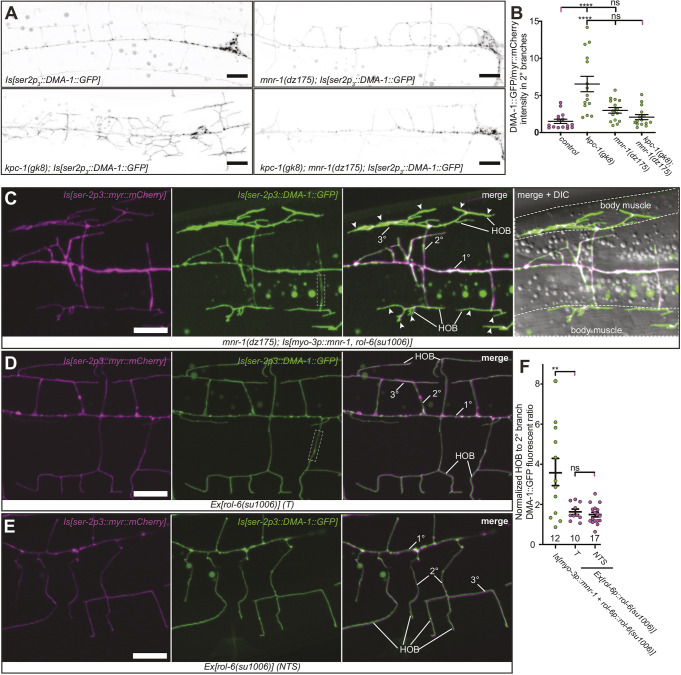
**MNR-1 is necessary and sufficient to retain DMA-1 on the membrane of somatosensory dendrites.** (A) Fluorescence micrographs (green channel) of animals of the indicated genotypes carrying transgenes for a functional DMA-1::GFP translational fusion {*qyIs369* [*Is(ser-2p3::DMA-1::GFP)*], green channel} and a myristoylated (myr) mCherry expressed in PVD {*wyIs581* [*Is(ser-2p3::myr::mCherry)*], red channel}. Scale bars: 10 µm. (B) Quantification of the DMA-1::GFP/myr::mCherry fluorescence ratio in 2° branches of the genotypes indicated. *n*=15 animals were analyzed for all genotypes. Error bars indicate the s.e.m. and statistical significance was calculated using one-way ANOVA with Šidák's correction for multiple comparisons. ns, not significant; ****P≤0.0001. (C-E) Fluorescence micrographs of animals of the indicated genotypes carrying transgenes expressing a functional DMA-1::GFP translational fusion {*qyIs369* [*Is(ser-2p3::DMA::GFP)*], green channel} and a myristoylated mCherry {*wyIs581* [*Is(ser-2p3::myr::mCherry)*], red channel} in PVD. In C, animals carry a transgene that drives expression of MNR-1 in muscle (*dzIs43* {*Is[myo-3p::MNR-1; rol-6::rol-6(su1006)]*}), whereas in D, animals carry a *rol-6* transgene to serve as a control for the integrated transgene in animals shown in C. Images in E represent a non-transgenic sibling (NTS) of the strain shown in D. HOB indicates higher order branches, arrowheads (C) indicate high concentration of DMA-1::GFP and the dashed white boxes (C,D) indicate regions for which DMA-1::GFP was quantified in secondary dendrites. Scale bars: 10 µm. (F) Quantification of fluorescence signals, expressed as the ratio between the normalized fluorescence signal of DMA-1::GFP in baobab 3° branches and 2° branches. The number of animals analyzed was: *Is[myo-3p::mnr-1+rol-6p::rol-6(su1006)]* (*n*=12), T (transgenic siblings) (*n*=10) and NTS (*n*=17). Error bars indicate the s.e.m. and statistical significance was calculated using one-way ANOVA with Šidák's correction for multiple comparisons. ns, not significant; **P≤0.01.

A hallmark of the *kpc-1* loss-of-function phenotype in PVD is the increased level of DMA-1 on the membrane. This has been suggested to result from diminished endocytosis of the DMA-1 receptor, based on colocalization in endocytic vesicles and co-immunoprecipitation experiments indicating that both KPC-1 and DMA-1 are part of a biochemical complex (Dong et al., 2016). DMA-1 accumulation on the neuronal surface is thought to result in increased adhesive forces, leading to dendrite trapping and reduced branch extension. An alternative scenario arose from our transgenic experiments with *mnr-1*: the similarity of *kpc-1* loss-of-function and *mnr-1* overexpression phenotypes raised the possibility that an excess of MNR-1 from the epidermis could retain DMA-1 on the plasma membrane of PVD dendrites. Interestingly, we previously found that the *mnr-1; kpc-1* double-null mutant more closely resembled the *mnr-1* null mutant than the *kpc-1* mutant (Salzberg et al., 2014), suggesting that *mnr-1* is epistatic or, in other words, that MNR-1 is required for the *kpc-1* mutant phenotype. We therefore analyzed the localization of a DMA-1::GFP reporter in single and double mutants of *mnr-1* and *kpc-1*. As previously reported (Dong et al., 2016), we detected a significant increase in diffuse DMA-1::GFP reporter levels in PVD in the absence of KPC-1 (Fig. 4A,B). Diffuse staining of the DMA-1::GFP reporter is considered to represent the cell membrane-bound fraction of the receptor (Taylor et al., 2015; Zou et al., 2015; Dong et al., 2016). Interestingly, the levels of the DMA-1::GFP reporter in *kpc-1* mutants were reduced to wild-type levels in the absence of MNR-1 (Fig. 4A,B), demonstrating that MNR-1 is necessary for increased membrane localization of the DMA-1::GFP reporter in the absence of the KPC-1 proprotein convertase.

We next asked whether MNR-1 was sufficient to increase DMA-1 levels in the PVD membrane. We used *mnr-1(dz175)* null mutant animals carrying (1) a transgene that ectopically expresses MNR-1 in muscle cells, (2) a transgene that expresses a DMA-1::GFP fusion in PVD and (3) a transgene that expresses myristoylated mCherry in PVD. We calculated the GFP (DMA-1) to mCherry (membrane) fluorescence ratio in tertiary branches, normalized to that in secondary branches, and compared it with the ratio in a control strain expressing only the DMA-1::GFP fusion protein and myristoylated mCherry in PVD (Fig. 4C-F; see Materials and Methods for details on quantifications). Exclusive expression of MNR-1 in the muscle results in higher-order branches reminiscent of baobab trees, as opposed to the stereotyped candelabra-like structures (Salzberg et al., 2013). We found that the muscle-derived, overexpressed MNR-1 resulted in much higher accumulation of the DMA-1::GFP reporter in tertiary branches of baobab-like dendrites compared with DMA-1::GFP accumulation in tertiary branches of wild-type animals, suggesting that MNR-1 is sufficient to recruit/retain the DMA-1::GFP reporter on the PVD membrane surface *in trans* (Fig. 4C-F; Fig. S3C,D). Collectively, these findings show that MNR-1 is necessary and sufficient to non-cell-autonomously control DMA-1 surface levels on PVD somatosensory dendrites.

### KPC-1 is localized to membrane compartments of higher-order branches and functions strictly cell-autonomously

**Fig. 5.**
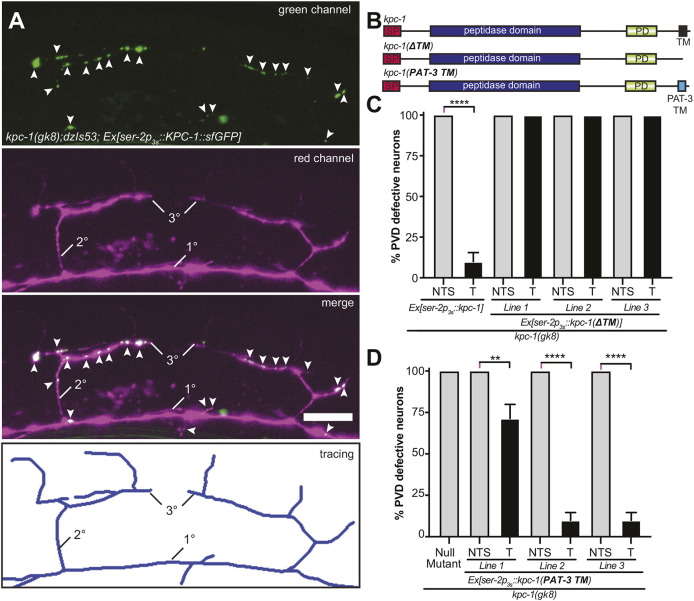
**A functional KPC-1 reporter is localized to higher-order branches and requires membrane attachment for function.** (A) Fluorescence micrographs of an animal transgenically expressing a functional KPC-1::sfGFP fusion specifically in PVD neurons (*dzEx1915*). PVD is visualized by *dzIs53 [Is(F49F12.4p::mCherry)].* The lower panel shows a tracing of the dendritic tree. Arrowheads indicate KPC-1::sfGFP in dendritic branches. Note that the KPC-1::sfGFP fusion also localizes to axonal tracks in the ventral nerve cord when expressed under control of a pan-neuronal promoter (Fig. S4B). Scale bar: 10 µm. (B) Schematics of KPC-1 constructs used in transgenic rescue experiments. SP, signaling peptide; PD, P domain; TM, transmembrane domain; PAT-3 TM, heterologous transmembrane domain of PAT-3. (C,D) Quantification of animals with defective PVD neurons in transgenic rescue experiments of *kpc-1(gk8)* null mutants with the indicated extrachromosomal transgenes. *n*=30 animals were analyzed for all genotypes. Error bars indicate the standard error of proportion and statistical significance was calculated using the Z-test with Bonferroni correction for multiple comparisons. T, transgenic animals; NTS, non-transgenic siblings. **P≤0.01, ****P≤0.0001.

Because MNR-1 is required for the *kpc-1-*mediated increase of DMA-1::GFP on the cell surface, we investigated the localization of a KPC-1 reporter in PVD. We found that a functional *KPC-1::sfGFP* reporter specifically expressed in PVD localized to higher-order dendritic branches of PVD neurons as well as to the PVD axonal compartment (Fig. 5A, Fig. S4A). We further found that the putative transmembrane domain of KPC-1 (Fig. S5) was necessary to direct PVD dendritic patterning because a construct lacking the predicted C-terminal transmembrane domain (S675 to S688) failed to rescue the *kpc-1* mutant phenotype, whereas a construct containing a heterologous transmembrane domain of PAT-3/β-integrin (PAT-3-TM) rescued the defects (Fig. 5B-D).

We next asked whether a *kpc-1* cDNA expressed in PVD neurons could rescue FLP dendrite morphogenesis defects in *kpc-1* mutant animals or, conversely, whether expression in FLP neurons rescued PVD dendrite morphogenesis defects. In both experiments, *kpc-1* functioned strictly cell-autonomously, i.e. it rescued the *kpc-1* mutant defects only in the neurons where it was expressed (Fig. S6). We conclude that, *in vivo*, KPC-1 is localized to a membrane compartment in higher-order dendrite branches and is therefore potentially in proximity to both MNR-1 expressed by epidermal cells and the DMA-1 receptor in PVD. We further suggest that owing to the strictly cell-autonomous function of *kpc-1*, a putative proteolytic target may not be diffusible.

### KPC-1 requires proteolytic activity beyond autocatalytic activation during PVD dendrite morphogenesis

**Fig. 6.**
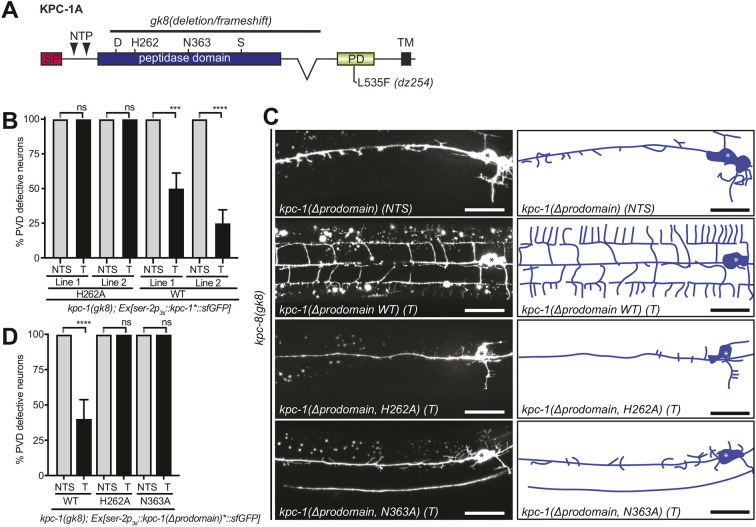
**KPC-1 requires catalytic activity beyond self-activation.** (A) Schematic of KPC-1 with alleles and amino acids important for catalytic activity indicated. SP, signaling peptide; PD, P domain; TM, transmembrane domain. (B) Quantification of transgenic rescue experiments of *kpc-1(gk8)* with versions of a full-length KPC-1::sfGFP fusion [kpc-1*::sfGFP, where ‘*’ represents the wild-type (WT) protein or H262A as noted] expressed under the PVD-specific *ser-2p3s* promoter. *n*=20 animals were analyzed for all genotypes. T, transgenic animals; NTS, non-transgenic siblings. (C) Fluorescence micrographs with schematics (right) of animals expressing the indicated transgenes in a *kpc-1(gk8)* null mutant background. PVD is visualized by the *wdIs52* transgene. The cell body is marked by asterisks. Scale bars: 20 µm. (D) Quantification of transgenic rescue experiments of *kpc-1(gk8)* with versions of a processed, mature KPC-1::sfGFP fusion [kpc-1(Δprodomain)*::sfGFP, where ‘*’ represents WT, H262A or N363A as noted] expressed under the PVD-specific *ser-2p3s* promoter. The number of animals analyzed was: *n*=24 (NTS, WT), 32 (T, WT), 40 (NTS, H262A), 40 (T, H262A), 40 (NTS, N363A) and 40 (T, N363A). For B,D, error bars indicate the standard error of proportion and statistical significance was calculated using the Z-test with Bonferroni correction for multiple comparisons. ns, not significant; ***P≤0.001; ***P≤0.001.

To better understand the proteolytic function of KPC-1 in PVD dendrite patterning, we conducted transgenic rescue experiments. We found that, consistent with previous studies, KPC-1 functions cell-autonomously in PVD neurons (Salzberg et al., 2014) and requires catalytic activity to pattern somatosensory dendrites (Fig. 6A,B) (Dong et al., 2016). Previous studies had further shown that expression of a mature, processed form of KPC-1 lacking the prodomain could effectively rescue *kpc-1* mutant phenotypes, suggesting that the catalytic activity of KPC-1 is required to autoactivate KPC-1 by cleavage of the prodomain (Dong et al., 2016). However, it remained unresolved whether the catalytic activity of KPC-1 is necessary beyond autocatalytic cleavage and activation. We confirmed that transgenic expression of the mature form of the KPC-1 (lacking the prodomain) in PVD rescued the *kpc-1* mutant phenotype in PVD (Fig. 6C,D) (Dong et al., 2016). However, a mature, processed form of KPC-1 carrying mutations in either of two conserved residues essential for convertase function (H262A or N363A in KPC-1) (Creemers et al., 1993) failed to rescue the *kpc-1* mutant phenotypes (Fig. 6C,D). This failure to rescue was not the result of obvious defects in protein stability or trafficking of mutant KPC-1, because a reporter fusion of mutant KPC-1 with a fluorescent protein [*KPC-1(H262A)::sfGFP*] expressed in PVD displayed protein expression levels and localization in PVD dendrites comparable with those of the analogous wild-type *KPC-1::sfGFP* reporter (Fig. S4C). Taken together, our experiments suggest that the convertase activity of KPC-1 is required beyond autocatalytic cleavage and imply the existence of additional substrate(s) for KPC-1 (other than itself) during PVD dendrite morphogenesis.

### MNR-1 and LECT-2 are regulated by KPC-1

**Fig. 7.**
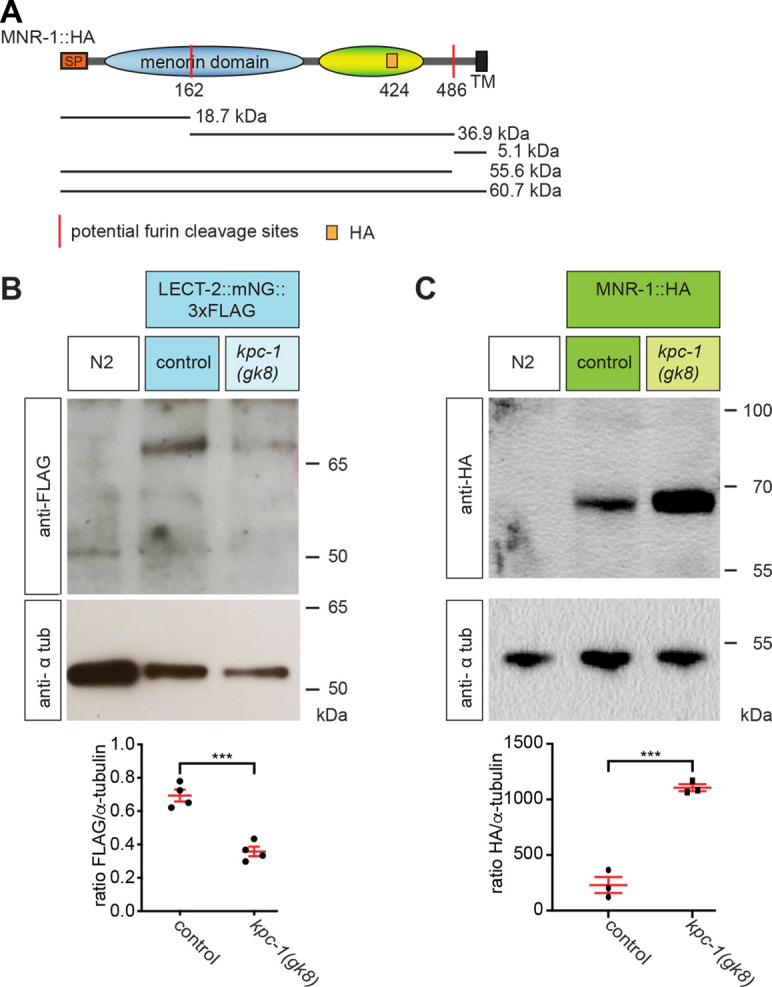
**MNR-1 is regulated by the KPC-1 proprotein convertase.** (A) Schematic of the putative MNR-1 protein originating from the endogenously modified *mnr-1* locus containing one HA tag between Y424 and Y425 (*dz293*). The computed molecular masses of theoretically possible cleavage products are indicated. SP, signal peptide; TM, transmembrane domain. (B) Western blot for LECT-2::3×FLAG and α-tubulin in *C. elegans* lysates. Experiments were performed on N2 wild-type animals or the genomically tagged LECT-2::mNeonGreen::3×FLAG (*dz249*; Diaz-Balzac et al., 2016) both in a wild-type and a *kpc-1(gk8)* null mutant background. The ratio of the LECT-2::mNeonGreen::3×FLAG and tubulin signal was calculated and the Kruskal–Wallis Test used to compute statistical significance (*n*=4 biological replicates). (C) Western blot against MNR-1::HA and α-tubulin in *C. elegans* lysates. Experiments were performed on N2 wild-type animals or the genomically tagged MNR-1::HA [*mnr-1(dz293) (mnr-1::HA)*] both in a wild-type and a *kpc-1(gk8)* null mutant background. The ratio of the MNR-1::HA and tubulin signal was calculated and the Kruskal–Wallis Test used to compute statistical significance (*n*=3 biological replicates). For B,C, molecular marker sizes are indicated in kDa on the right. ***P≤0.001.

Finally, we asked whether KPC-1 is required to regulate protein levels of the Menorin adhesion complex, including the putative cell adhesion molecules SAX-7, DMA-1 and MNR-1, and the secreted chemokine LECT-2. To this end, we used animals carrying transgenes or encoding genome-engineered versions of these proteins with immunotags. We found no obvious change in the amounts or banding patterns on western blots for SAX-7 in *kpc-1* null mutants compared with those for control animals (Fig. S7A), in spite of a conserved putative furin cleavage site in the fibronectin domain 3, which is important for SAX-7 function during PVD development (Dong et al., 2013; Salzberg et al., 2013). Similarly, we found no obvious changes in banding patterns for DMA-1, consistent with a prior report for DMA-1 (Dong et al., 2016) (Fig. S7B). In contrast, we found that the absolute amount of endogenously tagged LECT-2 was decreased, whereas the amount of MNR-1 was increased in *kpc-1* mutants compared with those in control animals (Fig. 7A-C). Although we could not detect obvious changes in banding patterns of MNR-1, our resolution may be insufficient to detect a possible change of 5 kDa resulting from cleavage from a predicted membrane-proximal furin cleavage site (Fig. 7A-C). Therefore, *kpc-1* does not obviously affect SAX-7 or the DMA-1 receptor but is involved in the regulation of the overall levels of MNR-1 and LECT-2 either directly or indirectly.

To further test how MNR-1 might be regulated by KPC-1, we devised a different assay. Previous studies showed that an N-terminally tagged version of MNR-1 (mCherry::MNR-1) accumulates in coelomocytes, a set of scavenger cells that uptake diffusible proteins from the pseudocoelom, raising the possibility that MNR-1 is shed from cell membranes or secreted (Salzberg et al., 2013). We therefore established a functional MNR-1 fusion with N-terminally fused tagRFP and C-terminally fused mNeongreen (Fig. S7C) and quantified accumulation of N-terminally tagged MNR-1 in coelomocytes. We discovered that the endocytic compartment of coelomocytes showed consistently higher red fluorescence from tagRFP (i.e. more N-terminally tagged MNR-1) in wild-type animals compared with that in *kpc-1* mutant animals (Fig. S7D,E). These observations were not the result of defective endocytosis in coelomocytes, because accumulation of mCherry in the coelomocytes of *kpc-1* mutant animals of a control strain was indistinguishable from that of wild-type animals (Fig. S6C). In contrast, we found no significant differences in the levels of the C-terminal mNeongreen fragment in the epidermis between control and *kpc-1* null mutant animals (Fig. S7D-G). We next measured the colocalization between the red and green fluorescence signals, i.e. between the N-terminal and the C-terminal MNR-1 fragments. To our surprise, the red (N-terminal) and green (C-terminal) fluorescent MNR-1 fragments in the epidermis almost perfectly colocalized in both wild-type and *kpc-1* null mutants (Fig. S7G). Lastly, biochemical experiments with western blotting using antibodies against the N-terminal tagRFP or the C-terminal mNeongreen revealed no obvious evidence for cleavage of the dually tagged MNR-1 transgene (not shown). We do not know the reason for this apparent discrepancy, but one possible explanation is that the amount of cleaved MNR-1 is too small to be detectable in the background of the overexpressed *tagRFP::MNR-1::mNeongreen* transgene. Alternatively, MNR-1 may not be a direct target of KPC-1. Regardless, taken together, our results show that the KPC-1 proprotein convertase directly or indirectly regulates the amounts of MNR-1 and LECT-2. Of note, based on the strictly cell-autonomous function of KPC-1 in PVD patterning (Salzberg et al., 2014; Dong et al., 2016) (this study), these observations suggest that KPC-1 from PVD dendrites regulates the non-cell-autonomously acting factors MNR-1 and LECT-2 from the epidermis and muscle, respectively, in a negative feedback loop of the Menorin pathway. Additional experiments will be required to determine the precise mechanisms of this regulation across cells.

## DISCUSSION

In this study, we establish a mutually antagonistic relationship between the KPC-1 proprotein convertase and the putative MNR-1 cell adhesion molecule. Genetically, phenotypes owing to partial- but not complete-loss-of-function mutations in *kpc-1* could be suppressed by partial but not complete loss of *mnr-1.* We further show that the previously known regulation of DMA-1 receptor localization in PVD by KPC-1 is dependent on MNR-1 in the epidermis. Additionally, we establish that enzymatic activity of KPC-1 is required beyond self-activation, implying the existence of a substrate for KPC-1 other than itself. Interestingly, we found elevated levels of MNR-1 as well as changed localization in *kpc-1* mutant animals, suggesting that the mutually antagonistic relationship between KPC-1 and MNR-1 is also true at the protein level. In addition, we found decreased levels of the muscle-derived factor LECT-2 in *kpc-1* mutants. Taken together, our findings suggest that KPC-1 functions autonomously in PVD neurons to regulate MNR-1 in the epidermis, which in turn regulates surface localization of the DMA-1 receptor on PVD. We propose that KPC-1 serves in a negative feedback loop as a functional off-switch for signaling by the Menorin adhesion complex during development.

It had previously been suggested that KPC-1 regulates PVD dendrite development through direct regulation of DMA-1 receptor accumulation on the membrane (Dong et al., 2016). It was further argued that the proteolytic activity of KPC-1 was required for self-activation, an event thought to be crucial for its function in endocytosis of DMA-1 (Dong et al., 2016). The endocytosis hypothesis for KPC-1 appeared to be supported by precedent of the mammalian convertase PCSK9 (pro-protein convertase subtilisin kexin type 9) in the clearance of low-density lipoprotein receptors from the cell surface (Cameron et al., 2006; Lagace et al., 2006). However, several arguments suggest that KPC-1 is functionally more similar to catalytically active furin than to the largely catalytically inactive PCSK9 convertase. First, KPC-1 has been shown to process insulin-like peptides in *C. elegans* (Hung et al., 2014), a function that is also performed by furin in mammals (Smeekens et al., 1992). Second, several structural aspects are also distinct between PCSK9 and KPC-1: (1) in contrast to PCSK9, the mature KPC-1 convertase neither requires nor associates with its prodomain (Dong et al., 2016) (Fig. S6); (2) KPC-1 contains a P domain, which, based on the *kpc-1(dz254)* allele we isolated, is functionally important, and likely requires calcium for its catalytic activity (Thacker and Rose, 2000); and (3) we demonstrate that KPC-1 requires a transmembrane domain for function (Fig. 5), a finding reminiscent of membrane-bound furin (Henrich et al., 2003) but distinct from the secreted PCSK9 (Piper et al., 2007). Taken together with our findings that KPC-1 requires the presence of the putative cell adhesion molecule MNR-1 to regulate DMA-1 localization, these observations argue against a simple role in endocytosis alone for KPC-1.

Our results raise the possibility that MNR-1 is shed from the epidermal membrane, whereas other factors of the MNR-1 pathway for PVD dendrite patterning, such as the epidermal cell adhesion molecule SAX-7 or the DMA-1 receptor itself, appear to be unaffected by loss of KPC-1 (Fig. S7). Unexpectedly, we also observed a decrease of LECT-2 in *kpc-1* mutants (Fig. 7B). A possible explanation is that an excess of available MNR-1 obscures putative binding sites for LECT-2 in SAX-7, thereby resulting in less binding opportunity and consequently degradation of LECT-2. Additional biochemical and genetic experiments will be required to resolve this question. Regardless, the catalytic activity of KPC-1 is required to regulate directly or indirectly the localization and the amount of the MNR-1 ligand *in trans*. It is therefore tempting to speculate that retention of DMA-1 by epidermal MNR-1 prevents endocytosis of the DMA-1 receptor in PVD and that KPC-1 negatively modulates this MNR-1 function, e.g. by promoting disassembly of the Menorin complex and the associated interactions. This KPC-1 function may be facilitated by DMA-1-dependent recruitment of the KPC-1 proprotein convertase, as shown previously (Dong et al., 2016). Adhesive signaling complexes face the conceptual challenge that their adhesive properties must be tightly regulated to allow coordinated growth during neural development (reviewed by Moreland and Poulain, 2022). Our genetic experiments reveal a tight antagonistic relation between MNR-1 and KPC-1. The phenotypes observed represent extremes of a continuum (Fig. 3): on one end of the spectrum, the *mnr-1* null mutant phenotype represents strongly reduced adhesiveness of PVD branches, whereas on the other end, the *kpc-1* null mutant phenotype represents excessive MNR-1 complex interactions and adhesiveness. Consistent with this interpretation, we find that both reducing and increasing the function of the Menorin pathway leads to defects in dendrite patterning. Therefore, fine-tuned control of the Menorin pathway is necessary to allow both extension and consolidation of developing dendrites. We propose that KPC-1 is a switch that locally controls adhesiveness of the Menorin pathway cell autonomously in PVD dendrites, by regulating the availability and or levels of the MNR-1 ligand in the epidermis *in trans*. Further experiments will be required to determine the precise mechanism (or substrate) by which KPC-1 convertase activity regulates dendrite morphogenesis in PVD neurons.

Experiments in heterologous systems have shown that proprotein convertases can process extracellular proteins, e.g. the glycoprotein reelin. However, this processing occurs *in cis*, i.e. with both furin and reelin functioning in the same cell (Kohno et al., 2015). In contrast, our observations could be consistent with a scenario in which KPC-1 can process/regulate substrates also *in trans*, i.e. on adjacent cells. Whereas, to our knowledge, there is no evidence in the literature of a proprotein convertase cleaving a substrate *in trans*, a similar scenario has been documented for ADAM metalloproteinases (Janes et al., 2005). Specifically, it was shown that ADAM10 associates with the ephrin receptor on one cell to cleave the cognate ephrin ligand *in trans* on another cell upon engagement of the ephrin ligand and the ephrin receptor, thereby providing negative feedback (Janes et al., 2005). Therefore, both proprotein convertases and ADAM family proteases, i.e. different classes of extracellular transmembrane proteases, appear to be associated with specific cell-surface receptors on the same cell to regulate a given signaling pathway. This may be a more general regulatory mechanism for signaling pathways, which provides cells receiving a signal from adjacent cells a powerful means for a feedback loop to negatively regulate the received signal.

## MATERIALS AND METHODS

### *C. elegans* strains and husbandry

*C. elegans* animals were grown on OP50 *Escherichia coli*-seeded nematode growth medium plates, usually at 20°C unless otherwise specified. Strains used in this work can be found in Table S1.

### Molecular biology and transgenesis

A complete list of transgenes and plasmids created for these studies is provided in Tables S2 and S3, respectively.

#### Testing *mnr-1* dosage levels effects in PVD dendrite branching

A fosmid containing the *mnr-1* locus including its regulatory regions (WRM618aD06) was injected at 1 or 20 ng/μl into *mnr-1(dz175); dzIs53* [*Is(F49H12.4::mCherry)*] animals with *myo-2p::mCherry* as an injection marker at 25 ng/μl and pBlueScript (Stratagene/Sigma-Aldrich) at 50 ng/μl. The same injection mix with 1 ng/μl of WRM618aD06 was injected into *dzIs53* control animals to assess putative *mnr-1* overexpression effects on PVD dendritic arbor morphology.

#### *rol-6* transgenesis

We previously generated the *dzIs49[Is(myo-3p::mnr-1)]* integrated strain that expressed MNR-1 in the body wall muscle of a *mnr-1* null mutant strain (Salzberg et al., 2013). The injection marker in this strain was *rol-6(su1006)*. We used this strain to quantify *DMA-1::GFP* expression levels when MNR-1 was expressed in the muscle. To control for the presence of the *rol-6(su1006)* injection marker in *dzIs49*, we injected otherwise isogenic animals without the integrated transgene mis-expressing MNR-1 in muscle with 40 ng/μl of *rol-6(su1006)* and 60 ng/μl of pBlueScript. Transgenic animals and non-transgenic siblings were used as controls for fluorescence intensity quantification and the acquisition of comparative images in Fig. 4 and Fig. S3.

#### Translational fusion reporter of KPC-1

*kpc-1* cDNA tagged at the C-terminus with *sfGFP* was cloned under control of a short version of the *ser-2prom3* promoter (which we term *ser-2p3s*) or the *rab-3p* promoter, and injected at 5 ng/μl into *dzIs53 [Is(F49F12.4p::mCherry)]* animals either with *myo-2p::mCherry* or *rol-6(su1006)* as injection markers at 25 ng/µl together with pBlueScript at 50 ng/μl. A mutagenized version of the *ser-2prom-3s::KPC-1::sfGFP* plasmid carrying the H262A substitution in KPC-1 was used to assess rescue and protein expression of this catalytically dead form of KPC-1.

#### Prodomain deletions of KPC-1

cDNA of *kpc-1* containing the signal sequence of KPC-1 but lacking its prodomain (H34 to R146) was cloned downstream of the *ser-2p3s* promoter. Two catalytically dead versions of this preprocessed, prodomain-less construct were generated by site-directed mutagenesis, including H262A (which is part of the catalytic triad) and N363A (which constitutes the oxyanion hole residue that is essential for catalytic activity in other proprotein convertases) (Creemers et al., 1993).

#### *mnr-1* fluorescent tagging

*mnr-1* plasmids doubly tagged with either N-terminal *mCherry* or *tagRFP* and C-terminal mNeonGreen were generated by digestion and ligation of individual N-terminal- and C-terminal-tagged *mnr-1* (cDNA) constructs (Salzberg et al., 2013). The final fusions *mCherry::mnr-1::mNeonGreen* or *tagRFP::mnr-1::mNeonGreen* were expressed under control of a *dpy-7p* epidermal promoter, and injected into *mnr-1(dz175)* animals at 1 ng/μl together with the *unc-122p::tagBFP* marker at 25 ng/μl, the *F49H12.4::tagBFP* marker at 25 ng/μl and pBlueScript at 50 ng/μl.

#### Additional *kpc-*1 and *mnr-1* transgenic experiments

A *dpy-7p::mnr-1* rescuing construct was injected directly into *kpc-1(gk333538); mnr-1(dz213)* double-mutant animals or used for site-directed mutagenesis to introduce the L135F substitution. The L135F mutant construct was injected into *kpc-1(gk333538); mnr-1(dz175)* double-mutant or *kpc-1(gk333538)* or *mnr-1(dz175)* single-mutant animals. All constructs were injected at a concentration of 5 ng/μl together with 25 ng/μl of *myo-3p::GFP* or *myo-3p::tagRFP* injection markers and 70 ng/μl of pBlueScript.

Plasmids carrying the *kpc-1* cDNA sequence under control of the *ser2prom3s* or the *mec-7p* promoters (Salzberg et al., 2014) were individually injected into *kpc-1(gk8); dzIs53; hmIs4* animals in which the defective dendritic arbors of PVD and FLP were visible. Constructs were injected at a concentration of 5 ng/μl either with 25 ng/μl of *myo-3p::tagRFP* or *ttx-3p::mCherry* injection markers, and 70 ng/μl of pBlueScript DNA.

### Genetic screen

*kpc-1(gk333538); dzIs53* animals were mutagenized with ethyl methane sulfonate and the progeny of cloned F1 animals was scored for suppression or enhancement of the *kpc-1* hypomorphic dendrite branch defects (Fig. S1). In a separate screen for modifiers of a *lect-2* hypomorphic phenotype (Rahman et al., 2022), an additional allele for *kpc-1* was identified. This new allele, *dz254*, results in a L535P missense mutation in the P domain of the proprotein convertase (Fig. 1; Fig. S5). *kpc-1(dz254)* mutant animals displayed shorter 1° branches and self-avoidance defects but not an increased number of 2° branches, compared with those of wild-type control animals (Fig. S2F,H). *dz254* is a hypomorphic allele of *kpc-1* because its phenotype was less severe compared with the phenotype of the presumptive *gk8* null allele and more severe when placed in trans with the *kpc-1(gk8)* deletion (Fig. S2F).

### Fluorescence microscopy and quantification

In all experiments, fluorescence images were captured in live *C. elegans* at the L4 larval stage using a Plan-Apochromat 40×/1.4 or 63×/1.4 objective on a Zeiss AxioImager Z1 Apotome. Worms were immobilized using 1 mM levamisole for 30 min and mounted on 4% agarose pads. At least 25 adult animals were scored per genotype. Optical sections were collected and, depending on the experiment, individual sections or maximum-intensity projections were used for further analysis.

For tracing, the region of interest (ROI) comprised 100 μm of the primary branch anterior to the PVD cell body. Morphometric analyses were conducted using the NeuronJ plugin of the FIJI software and branches were defined as follows: 2° dendritic branches as any neurite branching out of the primary dendrite in the ROI; 3° branches as any neurite coming from the tip of a 2° branch and located in close proximity to the sub-lateral nerve cords; and 4° dendritic branches as those originating from 3° dendritic branches and extending to the dorsal or ventral nerve tracts. The self-avoidance index was defined as the ratio of the number of gaps between adjacent 3° branches divided by the number of candelabras within the ROI.

#### DMA-1 fluorescence intensity

3° branch regions in the same candelabra/baobab and the corresponding 2° branch were used for DMA-1::GFP quantification in FIJI. Three different regions were quantified per candelabra or baobab (circle diameter, 3 μm, ROI), whereas one region was quantified for the corresponding 2° branch. Normalization was performed first by dividing the fluorescence signal in 3° branches by the fluorescence signal in the corresponding 2° branches (normalization of DMA-1::GFP expression levels in PVD) and then by dividing this ratio by the same ratio obtained for the same anatomical structures in the red channel (normalization of transgene expression levels in PVD). Red and green integrated density (ID) values were recorded for each ROI, avoiding regions with obvious green DMA-1::GFP puncta. For the mean background intensity value (MBV, mean gray value for the background), a circle with the same diameter (3 μm) was drawn adjacent to the ROI but outside of the branches and avoiding gut autofluorescence. The exposure time used for the red and green channels was 100 ms for both strains (EB3585 and EB3595); overexposed animals were excluded from the analysis and the fluorescent intensity ratio (FIR) was calculated using the following formula:

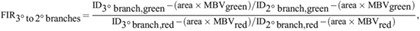
where ID is integrated density (product of area and mean gray value for the ROI), and MBV is the mean gray value (sum of the gray values of all the pixels in the selection divided by the number of pixels) for the background. ‘Green’ and ‘red’ represent calculations for the green and red channels.

The same procedure was performed for DMA-1::GFP fluorescence quantification in epistatic analyses, with few modifications: (1) three ROIs per 2° branch were quantified and (2) the arbitrary fluorescence intensity ratio was calculated as follows:

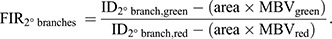


#### MNR-1 fluorescence intensity

L1 animals were synchronized by starvation overnight after egg preparation and, after 48 h on food, L4 animals were imaged. For quantification purposes, a single optical section in the mid-equatorial plane of the pair of coelomocytes located in the medial section of the animal was taken using a 40×/1.4 objective on a Zeiss AxioImager Z1 Apotome. The coelomocytes were encircled using the freehand selection tool in Fiji (ROI). Blue and red (or green) ID values were recorded for the exact same ROI. For the MBV value, a small circle, 3 μm in diameter, was drawn adjacent to the coelomocyte ROIs but avoiding gut autofluorescence. The exposure time used for the red, green and blue channels was 50 ms for strains carrying the *mCherry::mnr-1::mNeonGreen* construct (EB3599 and EB3600). The exposure time used for the red, green and blue channels was 100 ms, 100 ms and 50 ms, respectively, for strains carrying the *tagRFP::mnr-1::mNeonGreen* construct (EB3658 and EB3682). The fluorescence ratio was calculated using the following formula:

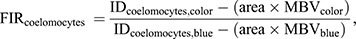
where ‘color’ represents calculations for the green or red channels and ‘blue’ represents calculations for the blue channels. The red channel (tagRFP) allowed visualization of N-terminal fluorescence for MNR-1, the green channel (mNeonGreen) allowed visualization of C-terminal fluorescence for MNR-1 and the blue channel (tagBFP) allowed visualization of a cytoplasmic reporter in the coelomocytes that was used for normalization of transgene expression.

### Modeling of KPC-1 and PCSK9 structures

A structural model of *kpc-1* was created using the I-TASSER server (Zhang, 2008; Yang et al., 2015), based on the crystal structure of human furin (PDB: 5JXG). The chosen model had a confidence score of −0.48 and an estimated TM-score (structural similarity) of 0.65. The pro-domain was removed from the sequence prior to simulation. The editing of the structure was performed using Chimera (Pettersen et al., 2004). The catalytic domain extends from N22 to K364 (shown in cyan, Fig.[Supplementary-material sup1]S5A), the P domain from H370 to V498 (shown in yellow), and the transmembrane domain from S529 to S542 (shown in purple). Wild-type residues corresponding to amino-acid substitutions in the mutated alleles used in this study are shown in red (R265 and L535). In the catalytic domain, the catalytic triad formed by the amino acids D221, H262 and S436 are shown in dark blue.

The structure of PCSK9 was downloaded from PDB (2PMW) and edited using Chimera. The pro-domain extends from T61 to Q152 (shown in orange; Fig.[Supplementary-material sup1]S5B), the catalytic domain from S153 to S447 (shown in cyan), and the V domain from G452 to H683 (shown in green). Note that contrary to other proprotein convertases, the prodomain of PCSK9 is cleaved only once and remains associated with the protease after cleavage.

### Immunoprecipitation and western blotting

For immunoprecipitation, five full plates of DMA-1::GFP transgenic worms were washed in RIPA buffer pH 7.0 (10 mM Tris-HCl, pH 7.0, 1% Triton X-100, 0.1% sodium deoxycholate, 0.1% SDS ,140 mM NaCl) and lysed for 15 min in a Biorupter water bath, as previously described for whole-worm protein extraction (Li and Zinovyeva, 2020). Post lysis, 20 µl of Protein A/G Plus Agarose beads (Santa Cruz Biotechnology, sc-2003) and 1 µl of undiluted anti-GFP (Roche, 11814460001) or anti-HA (Roche, 11867423001) antibodies were used to pull down DMA-1::GFP overnight at 4°C. For SAX-7::GFP::FLAG and LECT-2::mNeonGreen::FLAG western blots, ten and twenty gravid adult animals, respectively, were boiled in loading buffer and loaded directly into the gels. Gradient gels (4-12%, GenScript) were used for all experiments. To detect endogenous MNR-1::HA, 10 full plates of *C. elegans* were washed with HBS (25 mM HEPES, pH 7.5, 150 mM NaCl) and collected in a 15 ml Falcon tube. Following centrifugation at 1000 ***g*** for 5 min, the worm pellet was resuspended in HBS at 4× the volume of the pellet and 10% SDS at 1× the volume of the pellet. The suspension was sonicated with a probe sonicator at 40% power for 1 min with a 5* *s on/5* *s off cycle and centrifuged at 20,000 ***g*** for 10 min. The supernatant was combined with 4× SDS loading buffer and incubated at 60°C for 15 min, before loading onto gels for SDS-PAGE (10-20 µl for MNR-1::HA detection, 1-5 µl for α-tubulin detection). Standard SDS-PAGE and wet-transfer western blotting were followed with 5% milk used for blocking. Antibodies for western blots were used at the following concentrations: for anti-FLAG blots, 1:800 anti-Flag (Sigma-Aldrich, F1804) and 1:5000 HRP-conjugated anti-mouse IgG (Millipore, AP308P); for anti-GFP blots: 1:500 anti-GFP (Roche, 11814460001) and 1:5000 HRP-conjugated anti-mouse IgG (Millipore, AP308P); for anti-HA blots, 1:500 anti-HA (Roche, 11867423001) and 1:10000 HRP-conjugated anti-rat IgG (Invitrogen, 69-9520); and 1:5000 anti-α tubulin (12G10 monoclonal antibody from the Developmental Studies Hybridoma Bank created by the National Institute of Child Health and Human Development).

### Statistical analysis

Statistical tests were applied as described in each legend. Statistical significance of the number of 2° branches, 4° branches or self-avoidance index were calculated with one-way ANOVA using Sidak's correction for multiple comparisons. Statistical differences in self-avoidance and 4° branch number in *mnr-1* fosmid overexpression experiments were calculated by comparing transgenic and non-transgenic siblings using an unpaired, two-tailed *t*-test. DMA-1::GFP fluorescence ratio differences for epistasis analysis and for mis-expression of *mnr-1* experiments were calculated using a one-way ANOVA with Sidak's correction for multiple comparisons. For proportions, statistical significance was calculated using the z-test with Bonferroni correction for multiple comparisons where applicable. For comparisons of coelomocyte fluorescence intensity ratios, a non-parametric Mann–Whitney test was performed. All tests were performed using the Prism 7 Statistical Software suite from (GraphPad). Statistical significance is indicated as ns, not significant; *P≤0.05; **P≤0.01; ***P≤0.001; and ****P≤0.0001.

## Supplementary Material

10.1242/develop.201208_sup1 Supplementary information
