# Supplementary material for: Convertase-dependent regulation of membrane-tethered and secreted ligands tunes dendrite adhesion
Source: Development. 2023 Sep 18;150(18):dev201208. doi: 10.1242/dev.201208 (PMC10546877; doi:10.1242/dev.201208)
Supplement: Supplementary information [file develop-150-201208-s1.pdf]

A

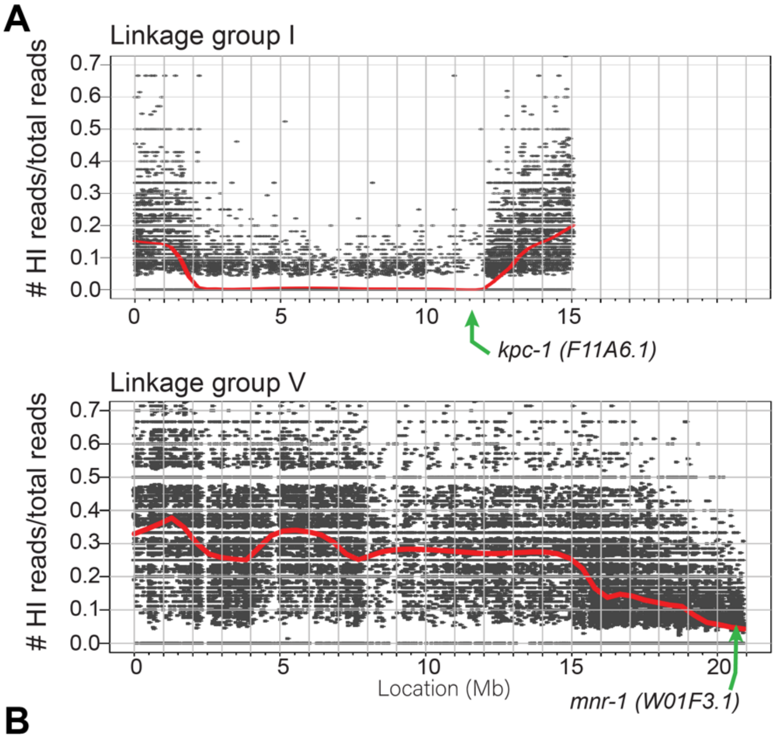

B

Mutant	Location	DNA change	a.a. change	Transcript ID	Gene Name
gk333538	LG-I 11678078	CGG>CAG	R265Q	F11A6.1	*kpc-1*
dz213	LG-V 20306260	TAT>TAA	Y63Stop	ZC15.1	*ZC15.1*
	LG-V 20357365	CAG>TAG	Q131Stop	K02E2.2	*grd-11*
	LG-V 20581165	GTG>GCG	V38A	F26F2.7	*F26F2.7*
	LG-V 20656966	CTC>TTC	L135F	W01F3.1a	*mnr-1a*
	LG-V 20656966	CTC>TTC	L81F	W01F3.1b	*mnr-1b*
	LG-V 20732758	GCG>GTG	A198V	W07A8.1	*srxa-1*

Fig. S1. Mapping of the extragenic suppressor allele *mnr-1(dz213)*.

A. Scatter plots of the ratio of the number of sequencing reads showing the CB4856 (Hawaiian) single nucleotide polymorphism (SNP) at a given position divided by the total number of sequencing reads at that same position. As expected, linkage is visible to both the *kpc-1* allele (*gk333538*, upper panel) and *mnr-1*(*dz213*, lower panel). Green arrows indicate the approximate positions of the genes on linkage groups I and V, respectively.

B. Table of polymorphisms identified in proximity of *kpc-1* and *mnr-1* on linkage group I and V, respectively. Because of its known involvement in dendrite patterning, we focused for transgenic rescue on *mnr-1* for the polymorphisms identified on linkage group V (see Figure S2A).

Development • Supplementary information

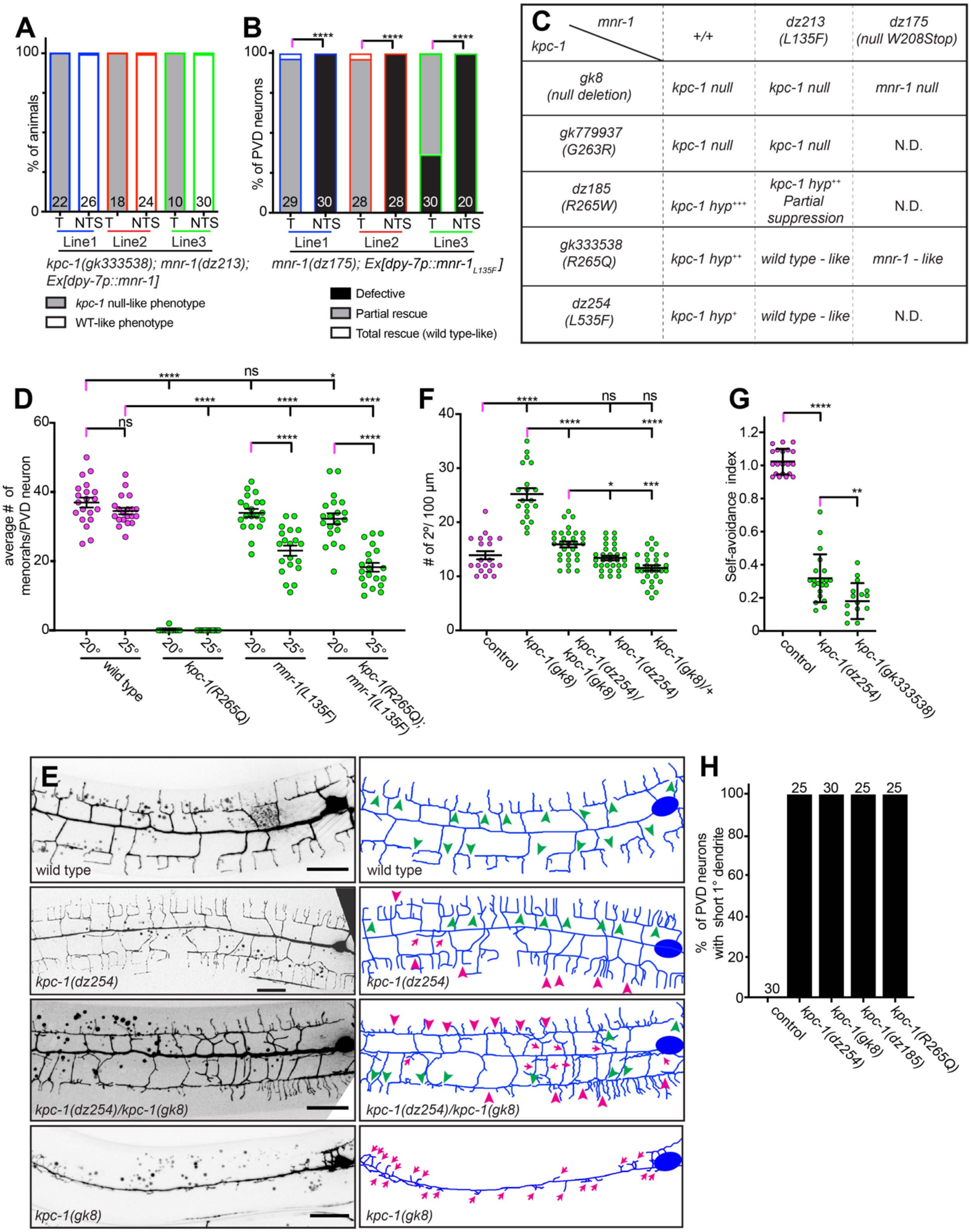

A
% of animals
22 26 18 24 10 30
T NTS T NTS T NTS
Line1 Line2 Line3
kpc-1(gk333538); mnr-1(dz213); Ex[dpy-7p::mnr-1]
kpc-1 null-like phenotype
WT-like phenotype
B
% of PVD neurons
29 30 28 28 30 20
T NTS T NTS T NTS
Line1 Line2 Line3
mnr-1(dz175); Ex[dpy-7p::mnr-1 L135F]
Defective
Partial rescue
Total rescue (wild type-like)
C
mnr-1
kpc-1
+/+
dz213 (L135F)
dz175 (null W208Stop)
gk8 (null deletion) kpc-1 null kpc-1 null mnr-1 null
gk779937 (G263R) kpc-1 null kpc-1 null N.D.
dz185 (R265W) kpc-1 hyp+++ kpc-1 hyp++ Partial suppression N.D.
gk333538 (R265Q) kpc-1 hyp++ wild type - like mnr-1 - like
dz254 (L535F) kpc-1 hyp+ wild type - like N.D.
D
average # of menorahs/PVD neuron
20° 25° 20° 25° 20° 25° 20° 25°
wild type
kpc-1(R265Q)
mnr-1(L135F)
kpc-1(R265Q); mnr-1(L135F)
F
# of 2°/ 100 µm
control
kpc-1(gk8)
kpc-1(gk8)/ kpc-1(dz254)
kpc-1(dz254)
kpc-1(gk8)/+
G
Self-avoidance index
control
kpc-1(dz254)
kpc-1(gk333538)
E
wild type
kpc-1(dz254)
kpc-1(dz254)/kpc-1(gk8)
kpc-1(gk8)
H
% of PVD neurons with short 1° dendrite
30 25 30 25 25
control
kpc-1(dz254)
kpc-1(gk8)
kpc-1(dz185)
kpc-1(R265Q)

Fig. S2. Reducing MNR-1/Menorin function alleviates partial *kpc-1/Furin* loss of function defects.

A. Quantification of transgenic rescue experiments of *kpc-1(gk333358); mnr-1(dz213)* double mutants with an epidermally expressed *mnr-1* cDNA under control of the *dpy-7p* promoter. Transgenic (T) animals of three independent lines and their non-transgenic siblings (NTS) are shown. The % of animals with a particular phenotype was determined and the number of animals is noted for each bar.

B. Quantification of transgenic rescue experiments of *mnr-1(dz175)* null mutant animals with an epidermally overexpressed *mnr-1(L135F)* mutant cDNA under control of the *dpy-7p* promoter. Transgenic (T) animals of three independent lines and their non-transgenic siblings (NTS) are shown. Full rescue was defined as PVDs with proximal menorahs (100 μm anterior to the cell body) with untangled and orthogonally located secondary, tertiary and at least two quaternary branches. Partial rescue was defined as PVDs with proximal menorahs (100 μm anterior to the cell body) composed of untangled and orthogonally located secondary and tertiary of variable length but with one or less quaternary branches. The % of animals with a particular phenotype is shown and the number of animals is noted for each bar. Statistical significance is shown as: **** $p \leq 0.0001$.

C. Summary of phenotypes of *kpc-1; mnr-1* double mutants of the indicated alleles. *kpc-1 hyp*; hypomorphic phenotype with primarily self-avoidance defects; *kpc-1 null*: null phenotype. N.D. not determined.

D. Quantification of the total number of 'menorahs' per animal in the genotypes and at the temperatures indicated. The number of animals analyzed was n=20 for all genotypes. Error bars indicate the standard error of the mean and statistical significance was calculated using one-way ANOVA with Šidák's correction for multiple comparisons. Statistical significance is shown as: ns not significant, * $p \leq 0.05$, ** $p \leq 0.01$, *** $p \leq 0.001$, **** $p \leq 0.0001$.

E. Fluorescent micrographs with schematics of the indicated genotypes. PVD is visualized by the *wdIs52* transgene. The cell body is marked by an asterisk. Green arrowheads indicate gaps

Development • Supplementary information

and red arrowheads defects in self-avoidance between neighboring 3º dendrites. Red arrows show short, immature 2º dendrites. Scale bar: 20 µm.

F. – H. Quantification of the number of 2º dendrites in a 100 µm segment anterior to the cell body (F, n=20), self-avoidance defects (expressed as self-avoidance index, G, n=20), and the % of PVD neurons with short 1º dendrites in the genotypes indicated (H, n as indicated above bars). Error bars indicate the standard error of the mean and statistical significance was calculated using one-way ANOVA with Šidák's correction for multiple comparisons. Statistical significance is shown as: ns not significant, * $p \leq 0.05$, ** $p \leq 0.01$, *** $p \leq 0.001$, **** $p \leq 0.0001$.

Development • Supplementary information

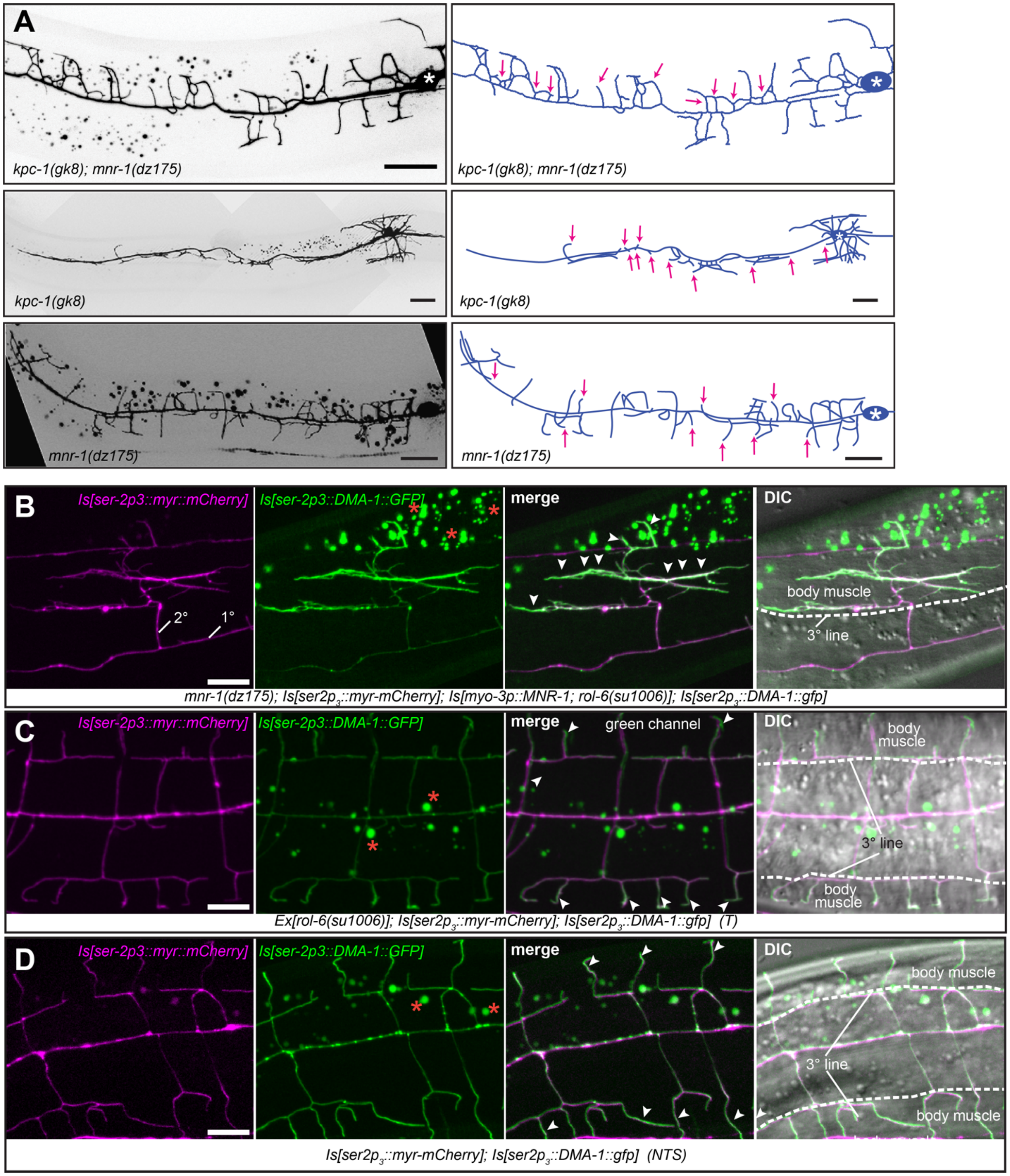
A
kpc-1(gk8); mnr-1(dz175)
kpc-1(gk8); mnr-1(dz175)
kpc-1(gk8)
kpc-1(gk8)
mnr-1(dz175)
mnr-1(dz175)
B
Is[ser-2p3::myr::mCherry]
Is[ser-2p3::DMA-1::GFP]
merge
DIC
2°
1°
body muscle
3° line
mnr-1(dz175); Is[ser2p3::myr-mCherry]; Is[myo-3p::MNR-1; rol-6(su1006)]; Is[ser2p3::DMA-1::gfp]
C
Is[ser-2p3::myr::mCherry]
Is[ser-2p3::DMA-1::GFP]
merge
green channel
DIC
body muscle
3° line
body muscle
Ex[rol-6(su1006)]; Is[ser2p3::myr-mCherry]; Is[ser2p3::DMA-1::gfp] (T)
D
Is[ser-2p3::myr::mCherry]
Is[ser-2p3::DMA-1::GFP]
merge
DIC
body muscle
3° line
body muscle
Is[ser2p3::myr-mCherry]; Is[ser2p3::DMA-1::gfp] (NTS)

Fig. S3. *mnr-1/Menorin* is epistatic to *kpc-1/furin* and required for *kpc-1*-dependent localization of DMA-1/LRR-TM.

A. Fluorescent micrographs with schematics of the indicated genotypes. PVD is visualized by the *wdIs52* transgene. The cell body is marked by an asterisk. Red arrows show short, immature 2º dendrites. Scale bar: 20 µm.

B. – D. Fluorescent micrographs (left three panels) and DIC (Differential Interference Contrast, right panels) of animals of the indicated genotypes carrying transgenes expressing a functional DMA-1::GFP translational fusion (*qyIs369* (*Is[ser-2p3::DMA::GFP]*), green channel) and a myristoylated myr.mCherry (*wyIs581* (*Is[ser-2p3::myr::mCherry]*), red channel) in PVD. In B. animals carry a transgene that drives expression of MNR-1 in muscle (*dzIs43,* Scale bar: 10 µm), whereas in C. animals are shown carrying a *rol-6* transgene to serve as a control for the integrated transgene in C.). D. presents a non-transgenic sibling (NTS) of the strain shown in C. Arrowheads indicate high levels of DMA-1::GFP localized to the plasma membrane and red asterisks autofluorescence. The tertiary line, i.e. the boundary between lateral epidermis and muscle is indicated by a dashed with line in the DIC panels.

Development • Supplementary information

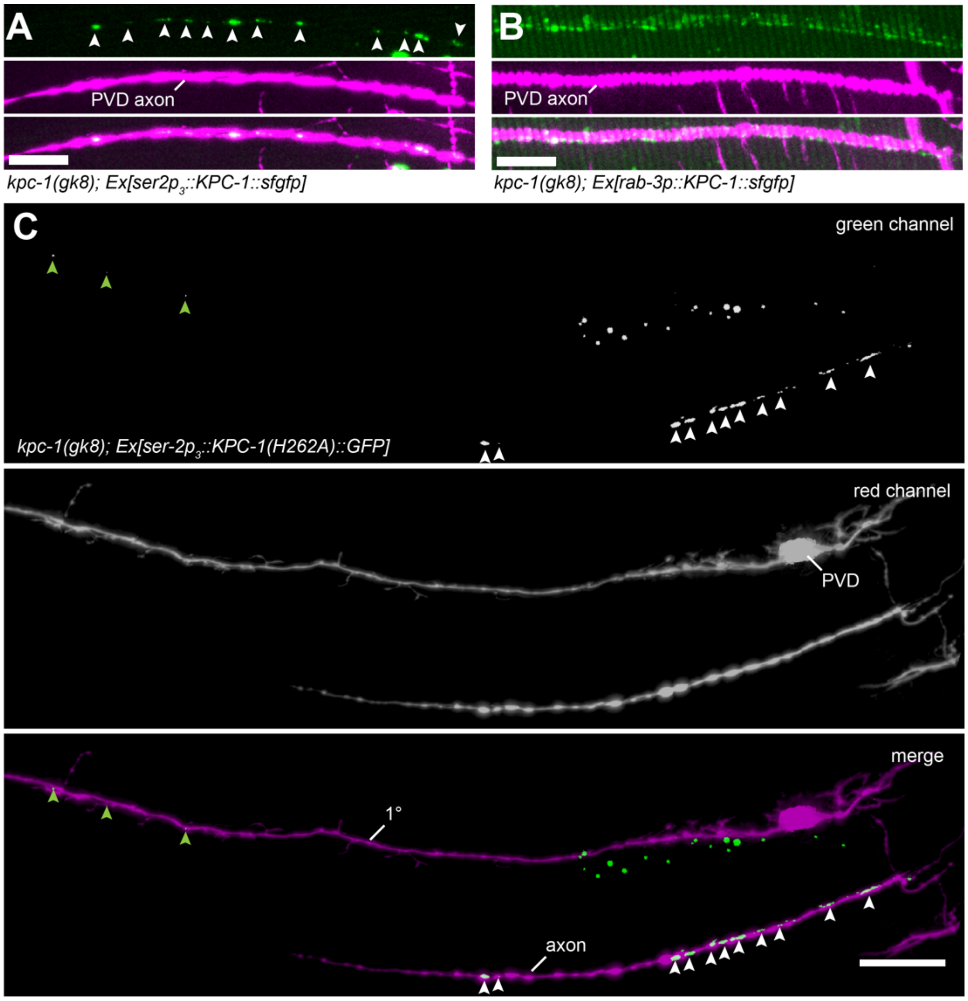

Fig. S4. A reporter for a catalytically dead KPC-1::sfGFP is localized normally in dendrites and axons.

A. -B. Fluorescence micrographs of an animal transgenically expressing a functional *KPC-1::sfGFP* fusion in PVD neurons (*dzEx1915*) (A) or all neurons (*dzEx1332*) (B). PVD is visualized by *dzIs53 (Is[F49F12.4p::mCherry])*. Note, that the *KPC-1::sfGFP* fusion also localizes to axonal tracks in the ventral nerve cord when expressed under control of a pan-neuronal promoter (B). White arrowheads indicate axonal staining. Scale bar is 20µm.

C. Fluorescence micrographs of an animal transgenically expressing a mutant *KPC-1(H262A)::sfGFP* fusion in PVD neurons (*dzEx1336*). Note that this strain also harbors the *him-5(ok1896)* allele, which has no effect on PVD morphogenesis. White arrowheads show axonal and green dendritic staining, respectively. Scale bar is 20 µm.

Development • Supplementary information

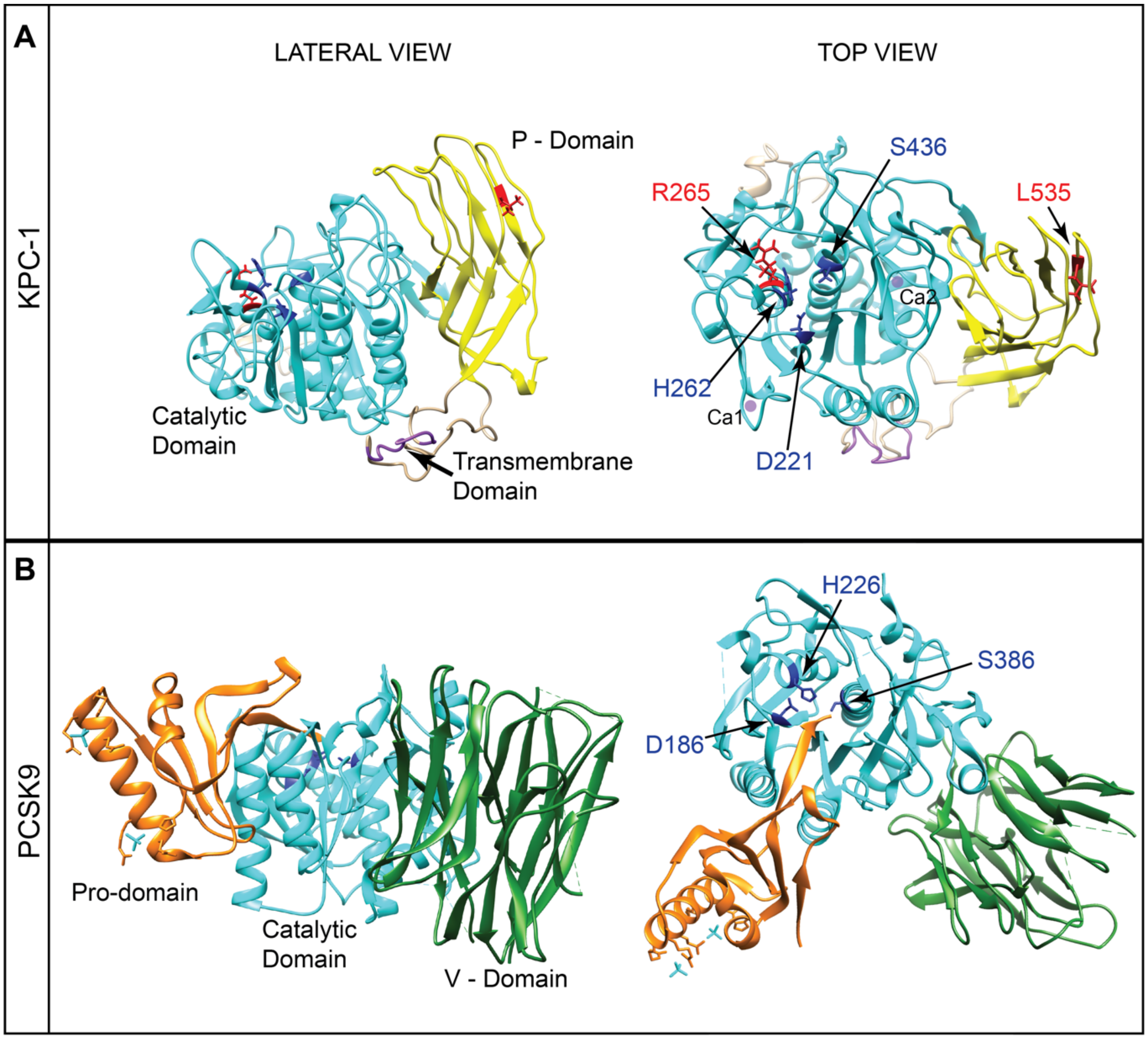

Development • Supplementary information

Fig. S5. Comparisons of putative molecular models for KPC-1/Furin and PCSK9.

Lateral and top views of the predicted KPC-1 structure (A, upper panel) and the PCSK9 crystal structure (B, lower panel). The structure of KPC-1 (A) was modeled using I-TASSER server (Zhang, 2008), based on the crystal structure of human Furin (PDB: 5JXG). The chosen model has a C-Score of -0.48 and an estimated TM-score (structural similarity) of 0.65. The pro-domain was removed from the sequence before the simulation. The structure was edited with Chimera (Pettersen et al., 2004). The catalytic domain extends from N168 to K510, the P domain from H516 to D644, and the transmembrane domain from S675 to S688. Mutations in the catalytic and P-domains are shown in red. Residues of the catalytic triad are shown as dark blue residues. The structure of the human proprotein convertase subtilisin kexin type 9 (B, PCSK9) was downloaded from PDB (2PMW) and edited using Chimera. The pro-domain extends from T61 to Q152, the catalytic domain from S153 to S447, and the V domain from G452 to H683. Catalytic triad residues are shown as dark blue residues.

Development • Supplementary information

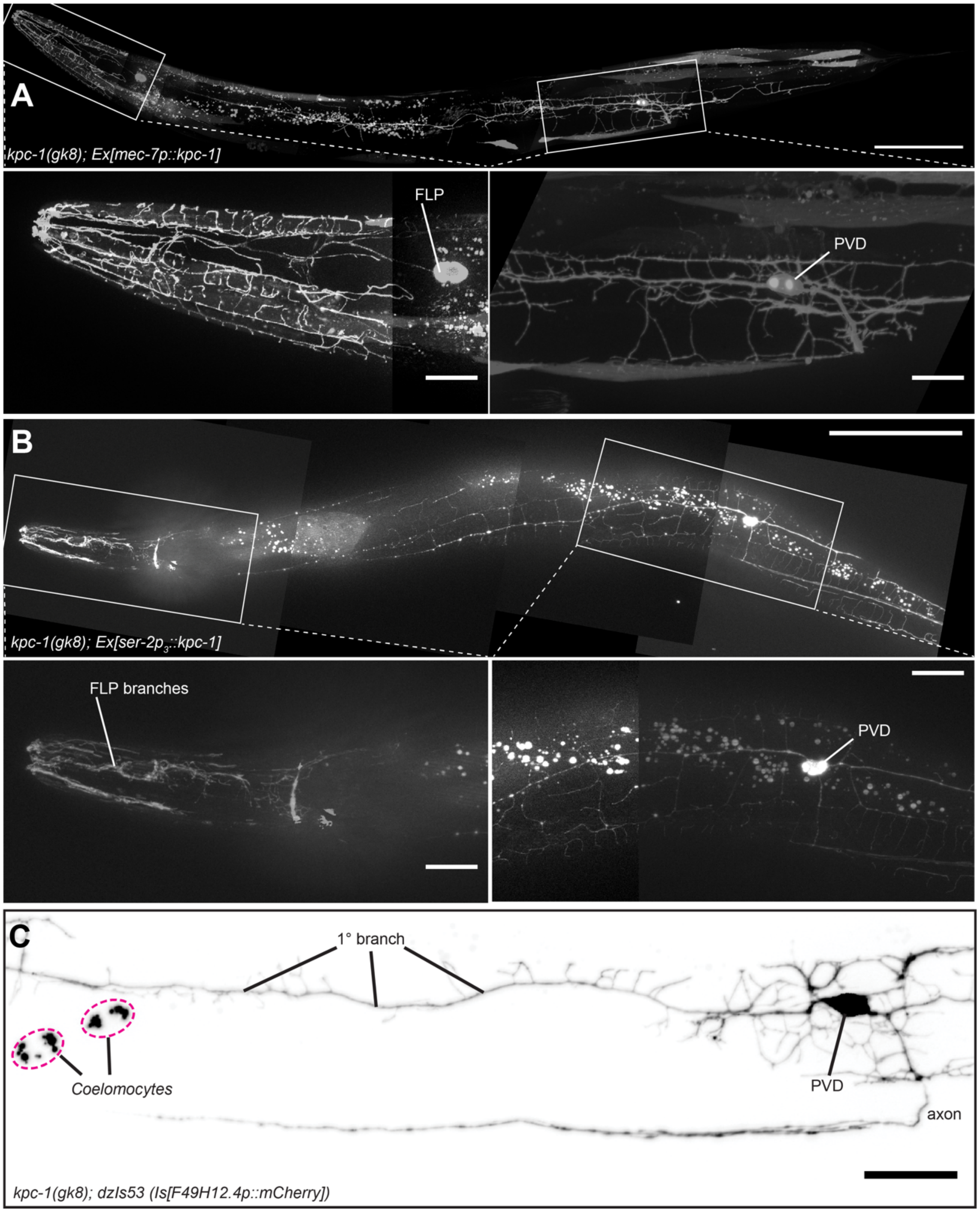

Development • Supplementary information

Fig. S6. *kpc-1/furin* functions strictly cell autonomously, and is therefore unlikely to process a diffusible target.

A. – B. Fluorescence micrographs of *kpc-1(gk8)* null mutant animals transgenically expressing a wild type *kpc-1* cDNA either under control of the *mec-7p* promoter in FLP (but not PVD) (A) or under control of the *ser-2p3* promoter in PVD (but not in FLP) (B). Note that expression in FLP rescues the mutant phenotype in FLP but not PVD (A), whereas vice versa expression in PVD but not FLP rescues the mutant phenotype in PVD but not FLP (B). Insets show FLP and PVD as denoted. Scale bars: 100 and 20 µm (insets).

C. Fluorescence micrographs of a *kpc-1/furin* mutant animals in the *dzIs53* transgenic background in which PVD is visualized by expression of mCherry in PVD. Note that some amount of mCherry is secreted in these animals and taken up by coelomocytes. Scale bar: 20 µm.

Development • Supplementary information

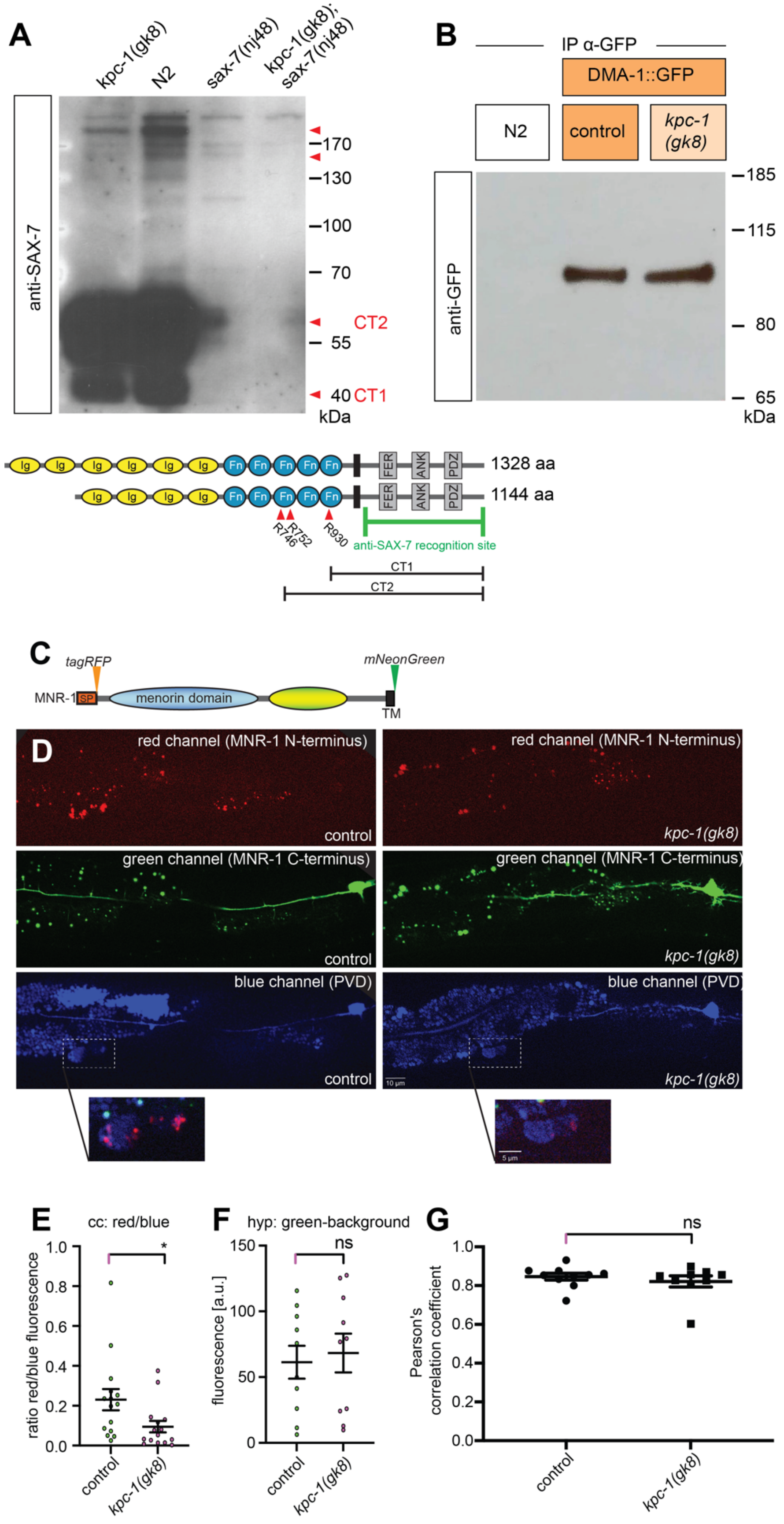

Development • Supplementary information

Fig. S7. SAX-7/L1CAM, DMA-1::GFP, and LECT-2/Chondromodulin II are not visibly affected by loss of *kpc-1/furin.*

A. – B. Western blots against SAX-7 (A) and DMA-1::GFP (B) in *C. elegans* lysates of the genotypes indicated, either directly (A), or after precipitating with an anti-GFP (B). Ladder is marked in kilodaltons (kDa). Experiments were performed on N2 wild type animals or tagged versions both in a wild type and a *kpc-1* null mutant background as follows: in wild type background (for A), or the integrated transgene DMA-1::GFP (*qyIs369* ,) (for B). A molecular marker with sizes indicated in kDa is shown on the right. Schematics that indicate predicted cleavage sites in SAX-7 are shown that result in two distinct cleavage products (CT1, C-terminal fragment 1, CT2, C-terminal fragment 2) in (A); red arrowheads indicate the cleaved forms as well as the uncleaved SAX-7S and SAX-7L forms.

C. Schematic of a functional transgene of MNR-1/Menorin construct where the N-terminus and C-terminus are fused in frame to tagRFP and mNeonGreen as indicated. TM: transmembrane domain.

D. Fluorescent micrographs of animals of the indicated genotypes. All animals carry the following transgenes: *dzIs125* (integrated *dzEx1894*), which labels PVD and the coelomocytes in blue and expresses the dually tagged *tagRFP::MNR-1::mNG* in the epidermis), and *wdIs52* (which labels PVD in green). Note that there is some GFP bleed through in the BFP channel. Inset (arrow) show coelomocytes magnified.

E. – F. Quantification of the fluorescence ratio (red (N-terminus)/blue (background)) in coelomocytes (E) and (green (C-terminus minus background) in the epidermis (G), respectively. The number fo animals analyzed was n=15 for E and n=10 for F. Statistical significance was calculated using the Mann-Whitney test and is indicated as ns, not significant; * $p \leq 0.05$.

G. Pearson's correlation efficients are shown between red (N-terminus) and green (C-terminus). Note that there is almost perfect colocalization.

Development • Supplementary information

Table S1. Strains used in this study.

Click here to download Table S1

Table S2. Extrachromosomal lines generated in this study.

Extrachromosomal array	Description
dzEx1957-59	*Ex[dpy-7p::mnr-1 + myo-3p::gfp]*
dzEx1979	*Ex[dpy-7p::mnr-1(L135F) + myo-2p::mCherry]*
dzEx1851-53	*Ex[WRM618aD06 (1ng/ul) + myo-2p::mCherry]*
dzEx1854-56	*Ex[WRM618aD06 (20ng/ul), myo-2p::mCherry]*
dzEx1976-78	*Ex[dpy-7p::mnr-1 (10ng/ul) + myo-2p::mCherry]*
dzEx1884	*Ex[rol-6(su1006)]:*
dzEx1865	*Ex[ser-2p3::kpc-1::sfgfp + myo-2p::mCherry]*
dzEx1915	*Ex[ser-2p3s::kpc-1.sfgfp+myo-2p::mCherry]*
dzEx1332	*Ex[rab-3p::kpc-1.sfgfp+pRF4]*
dzEx1916	*Ex[rab-3p::kpc-1::sfgfp + myo-2p::mCherry]*
dzEx1931-33	*Ex[ser-2p3s::kpc-1(-TMD) + myo-3p::tagRFP]*
dzEx1934-36	*Ex[ser-2p3s:kpc-1(-TMD +PAT-3TMD) + myo-3p::tagRFP*
dzEx1937-39	*Ex[ser-2p3s::kpc-1 + myo-3p::tagRFP]*
dzEx1336	*Ex[ser-2p3s::kpc-1(H262A).sfgfp + pRF4*
dzEx1331	*Ex[ser-2p3s::kpc-1.sfgfp+pRF4]*
dzEx1920-22	*Ex[ser-2p3s.(Δprodomain)kpc-1 + myo-2p::mCherry]*
dzEx1924-26	*Ex[ser-2p3s.(-prod)kpc-1(H262A) + myo-2p::mCherry]*
dzEx1927-29	*Ex[ser-2p3s.(-prod)kpc-1(N363A) + myo-2p::mCherry]*
dzEx1914	*Ex[ser-2p3s::kpc-1 + ttx-3p::mCherry]*
dzEx1911	*Ex[mec-7p::kpc-1 + myo-3p::tagRFP]*
dzEx1894	*Ex[dpy-7p::tagRFP::mnr-1::mNeonGreen + unc-122p::tagBFP + F49H12.4p::tagBFP]*
dzEx1964-66	*Ex[dpy-7p::mnr-1(L135F) + myo-3p::tagRFP]*
dzEx1967-69	*Ex[dpy-7p::mnr-1(L135F) + myo-3p::tagRFP]*

Table S3. Plasmid construction and primers.

Click here to download Table S3

Development • Supplementary information

REFERENCES

Oren-Suissa, M., Hall, D. H., Treinin, M., Shemer, G. and Podbilewicz, B. (2010) 'The fusogen EFF-1 controls sculpting of mechanosensory dendrites', *Science* 328(5983): 1285-8.
Pettersen, E. F., Goddard, T. D., Huang, C. C., Couch, G. S., Greenblatt, D. M., Meng, E. C. and Ferrin, T. E. (2004) 'UCSF Chimera--a visualization system for exploratory research and analysis', *J Comput Chem* 25(13): 1605-12.
Salzberg, Y., Coleman, A. J., Celestrin, K., Cohen-Berkman, M., Biederer, T., Henis-Korenblit, S. and Bülow, H. E. (2017) 'Reduced Insulin/Insulin-Like Growth Factor Receptor Signaling Mitigates Defective Dendrite Morphogenesis in Mutants of the ER Stress Sensor IRE-1', *PLoS Genet* 13(1): e1006579.
Smith, C. J., Watson, J. D., Spencer, W. C., O'Brien, T., Cha, B., Albeg, A., Treinin, M. and Miller, D. M., 3rd (2010) 'Time-lapse imaging and cell-specific expression profiling reveal dynamic branching and molecular determinants of a multi-dendritic nociceptor in C. elegans', *Dev Biol* 345(1): 18-33.
Zhang, Y. (2008) 'I-TASSER server for protein 3D structure prediction', *BMC Bioinformatics* 9: 40.
