# Supplementary material for: Convertase-dependent regulation of membrane-tethered and secreted ligands tunes dendrite adhesion
Source: Development. 2023 Sep 18;150(18):dev201208. doi: 10.1242/dev.201208 (PMC10546877; doi:10.1242/dev.201208)
Supplement: Supplementary file 1 [file develop-150-201208_review_history.pdf]

Convertase-dependent regulation of membrane-tethered and secreted ligands tunes dendrite adhesion

Nelson J. Ramirez-Suarez, Helen M. Belalcazar, Maisha Rahman, Meera Trivedi, Leo T.H. Tang and Hannes E. Bülow
DOI: 10.1242/dev.201208

Editor: Steve Wilson

Review timeline

Original submission:	17 August 2022
Editorial decision:	12 September 2022
First revision received:	23 June 2023
Editorial decision:	25 July 2023
Second revision received:	31 July 2023
Accepted:	1 August 2023

Original submission

First decision letter

MS ID#: DEVELOP/2022/201208

MS TITLE: Convertase-dependent regulation of a membrane tethered ligand in trans tunes dendrite adhesion

AUTHORS: Nelson J Ramirez-Suarez, Helen M Belalcazar, Maisha Rahman, Meera Trivedi, Leo T.H. Tang, and Hannes E Buelow

I have now received all the referees' reports on the above manuscript, and have reached a decision. The referees' comments are appended below, or you can access them online: please go to BenchPress and click on the 'Manuscripts with Decisions' queue in the Author Area.

As you will see, the referees express considerable interest in your work, but have some significant criticisms and suggestions for improving your manuscript. If you are able to revise the manuscript along the lines suggested, I will be happy to receive a revised version of the manuscript. Please also note that Development will normally permit only one round of major revision. If it would be helpful, you are welcome to contact us to discuss your revision in greater detail. Please send us a point-by-point response indicating your plans for addressing the referee's comments, and we will look over this and provide further guidance.

Please attend to all of the reviewers' comments and ensure that you clearly highlight all changes made in the revised manuscript. Please avoid using 'Tracked changes' in Word files as these are lost in PDF conversion. I should be grateful if you would also provide a point-by-point response detailing how you have dealt with the points raised by the reviewers in the 'Response to Reviewers' box. If you do not agree with any of their criticisms or suggestions please explain clearly why this is so.

© 2023. Published by The Company of Biologists under the terms of the Creative Commons Attribution License (https://creativecommons.org/licenses/by/4.0/).

Reviewer 1

Advance summary and potential significance to field

In this paper, Ramirez-Suarez, Buelow and colleagues explore how balance between the proprotein convertase KPC-1/Furin and the adhesion molecule MNR-1/Menorin regulates dendrite arborization of the multidendritic PVD neurons in C. elegans. The highly structured PVD dendrite morphology makes it a perfect system to examine molecular signals that instruct such an elaborate neuronal architecture. It has long been a puzzle how a proprotein convertase regulates dendrite morphologies, as it is widely assumed that substrates of this proprotein convertase are within the dendrites/neurons. Now, in the current study, the authors entertain an intriguing idea that KPC-1 regulates an adhesive ligand in trans in the substratum over which the dendrites grow. They leverage the power of nematode genetics to reveal that the PVD phenotypes of a kpc-1 hypomorphic mutant, previously thought to be due to increased DMA-1 receptor levels on the dendrite membrane, could be ameliorated by decreasing MNR-1 levels. They went on to address whether KPC-1 can function as a secreted protease, and whether it regulates the levels of putative adhesive ligands, in particular MNR-1. Most of the genetic experiments were well designed and rigorously executed, and the interpretation of the data was mostly clear and convincing. However experimental evidence supporting that KPC-1 regulates MNR-1 level in a cell-non-autonomous fashion is controversial (see below). The following issues need to be addressed by additional experiments or revision of the manuscript, before the paper could be considered for publication at Development.

Comments for the author

Major points:
1. The authors should elaborate more in the Introduction how the distribution of MNR-1/SAX-7 ligand complex in the hypodermis shapes the stereotyped dendritic arborization of PVD. In particular, the tertiary dendrites of PVD grow along the sublateral boundaries of the body wall muscles. This spatial relationship between the PVD dendrites and the neighboring muscles or hypodermal cells needs to be explained in more details, so that the readers can understand experiments in Fig. 4C-4E. They are also advised to introduce the spectrum of phenotypes seen in kpc-1 and mnr-1 mutant alleles of various strength, from self-avoidance defects to stalled branch growth or even a complete collapse of the dendritic arborization.

2. The most significant problem of this paper lies in Fig. 7, where the authors claim that KPC-1 regulates the overall levels of MNR-1. If KPC-1 cleaves MNR-1 at aa486 to produce a 55.6 kDa fragment, one would predict that this fragment will decrease in the kpc-1 null mutant. The increased level of MNR-1 in the kpc-1 mutant is opposite to this prediction, although it is consistent with mnr-1 mutation being epistatic to the kpc-1 mutation (Fig. S2C). To settle this issue one may need to mutate the putative KPC-1 sites of MNR-1, but this is not trivial. In Fig. S7C, the level of LECT-2 seems to be significantly decreased in the kpc-1 mutant. An LECT-2/tubulin ratio should be provided before one concludes whether the level of LECT-2 is affected in the kpc-1 mutant. In Fig. 7D and 7E, it seems that signals of tagRFP and NeonGreen were significantly dimmer in the kpc-1 mutant, compared to those in the wild type. This could affect the signal intensity of tagRFP in the coelomocytes – this is not a problem of the coelomocytes in the kpc-1 mutant, but rather the possibility that kpc-1 regulates mnr-1 expression should be excluded. As the authors claim that KPC-1 acts in trans to regulate MNR-1 levels in a different tissue, these issues should be rigorously addressed. Depending on the results of additional experiments, “in-trans” in the title of the paper may need to be tuned down.

3. Fig. 2E-2G: There are a couple of problems here. (1) Please explain the rationale of analyzing kpc-1(gk333538)/kpc-1(gk8); mnr-1(dz213)/mnr-1(dz175).
(2) Data of the control, kpc-1(gk333538) and kpc-1(gk333538); mnr-1(dz213) in 2F seem to be re-used in Fig. 2G. If so, Fig. 2F should be removed, as Fig. 2G contains all the data. (3) In Fig. 2G, mnr-1(dz13) should be mnr-1(dz213).

4. Fig. 3: In 3E, it would be nice if the PVD defects of the kpc-1(gk333538) could be directly compared with those of low-dose MNR-1 overexpression in the wild-type background. In 3C, an

© 2023. Published by The Company of Biologists under the terms of the Creative Commons Attribution License (https://creativecommons.org/licenses/by/4.0/).

image of kpc-1(gk8) was shown. However, in 3F the data of kpc-1(dz254) were presented. The reason for this is apparently that kpc-1(gk8) is so severe that characterization of self-avoidance is basically impossible. If so, the authors should explain this in more details, and make it consistent to present the same kpc-1 mutant for the image and quantification.

5. Fig. 4C-4E: The layout of these panels/images should be significantly modified. The pseudocolor coding is a bit confusing, especially in 4D and 4E. The brightness and contrast of these images need to be improved. Fig. 5A looks much better. (1) For all monochromic channels (green/red), please annotate them as “ser2p3::DMA-1::GFP” and “ser2p3::myr::mCherry”, respectively. (2) Please add dual-color merge images for DMA-1/PVD that allow the readers to see DMA-1 reruitment to the tertiary dendrites in 4C but not in 4D or 4E. (3) The authors should show MNR-1 expression in the muscles (fused to tagBFP, etc). From the current images, it is hard to imagine whether the tertiary dendrites are trapped on the muscle surface, or whether MNR-1 on the muscle membrane is redistributed by binding to DMA-1 on the PVD dendrites. Or at least the muscle borders should be shown by DIC as in Fig. S3B-3D.

6. Page 12: “We therefore asked whether … can act locally or at a distance….. substrate can function in a diffusible form or not”. I suggest the authors rephrase this statement, as it is both vague and confusing in what the experiment is trying to address. The argument and the experimental design here are also perplexing. The results in Fig. S4 basically show that kpc-1 acts cell-autonomously, which is consistent with the data that a secreted KPC-1 is non-functional. This experiment (FLP/PVD cross-rescue) does not add much to our understanding of the KPC-1 mechanisms. If the authors would like to keep these data, I suggest that they be combined with the cell-autonomous rescue experiments of PVD (Fig. 5) but not as a separate section.

Minor points:
1. Please add line numbers to help the review process go more efficiently.
2. Abstract: “Lastly, we find increased amounts of MNR-1….” Please clarify under what conditions/genetic backgrounds is increased MNR-1 in the coelomocytes detected.
3. I suggest the authors to replace “semblance” with “similarity” (Page 4, 10).
4. In Fig. 4B, please use arrows to highlight the secondary PVD dendrites where levels of DMA-1::GFP were quantified.
5. The y-axis labels of Fig. 5C, 5D, 6B, 6D should be “% defective PVD neurons”?
6. Fig. S4 was cited much later than Fig. S5. The authors may want to re-order the supplemental figures so that they match the order of citation in the paper.
7. Are KPC-1 and DMA-1 colocalized or in proximity to each other on the tertiary PVD branches? The experiment to test this should be straightforward.
8. Fig. S5B: Please show the localization of KPC-1(H262A) in the tertiary PVD dendrites. The current images only show KPC-1(H262A) in the axon and the primary dendrite, but it is the tertiary dendrite where KPC-1 is supposed to act to regulate dendrite morphogenesis.
9. Page 15: “SAX-1/L1CAM” should be “SAX-7/L1CAM”.

Reviewer 2

Advance summary and potential significance to field

The paper by Ramirez-Suarez et al., concerns mechanisms of dendritic patterning expanding on a previously established adhesion complex which serves to pattern a pair of arborized neurons in the nematode Caenorhabditis elegans. The authors provide genetic evidence using partial loss-of-function alleles for the regulation of an adhesion pairing between the skin expressed MNR-1/Menorin and the growing dendrite expressing DMA-1/LRR-TM on the sensory neuron PVD (with some evidence for a second neuron, FLP). This interaction is regulated by KPC-1/Furin, which is required on the neuron membrane in its catalytically active form. Loss of kpc-1 is correlated with decreased accumulation of, possibly cleaved, MNR-1 in coelomocytes. The authors suggest that cleavage or some other regulation in trans by dendritic-anchored KPC-1 on hypodermal MNR-1 is responsible for anchoring the dendritic DMA-1 to the membrane (bound by MNR-1), or, conversely, its removal from the membrane.

© 2023. Published by The Company of Biologists under the terms of the Creative Commons Attribution License (https://creativecommons.org/licenses/by/4.0/).

Comments for the author

The paper is well-written, although it could benefit from a clear model depiction, more careful dose-response analysis of different rescue constructs and additional overexpression experiments and ectopic-tissue expression experiments. Lastly, time-lapse imaging may provide valuable evidence for protein localization and/or cleavage, in particular if coupled with tissue-specific expression of kpc-1 as well as temperature shifts of the mnr-1 (dz213) temperature-sensitive allele described.

In order to provide sound evidence for the trans interaction which is inferred by the genetic results, we advise the following experiments:
The authors can use the dual-marked MNR-1 (N-terminus tagRFP, C-terminus mNeonGreen) as a key reagent for showing potential cleavage and altered localization under the various genetic backgrounds. This strain can be used for the following: a) time lapse imaging to follow potential shedding of the extracellular fragment of MNR-1, either in vesicles or in another form; b) repeating the IP experiments in the wildtype and kpc-1 mutant using antibodies against RFP and against mNeonGreen, in order to try and detect the two fragments of the protein; c) combining the timelapse mentioned above under the background of the catalytically-dead KPC-1(H262A), or wild-type KPC-1 expressed from a non-PVD promotor.
Additional experiments may involve a more precise characterization of the inter-dependence of MNR-1 and KPC-1 amounts by coinjecting kpc-1 and mnr-1 at similar levels to determine whether the amounts may be balanced even in an over-expression like state.

The paper requires rewriting to help readers follow the various alleles and their phenotypes. While a simple model is presented in figure 3G, it remains difficult to follow the relationship between the various alleles presented. Additionally, the text, figures, and figure legends need a careful reading for typos as well as missing and/or duplicated information.

Detailed suggestions –
major comments:
While the genetic evidence presented nicely supports the effect of kpc-1 on the hypodermally-expressed mnr-1, and is pinpointed to depend on the catalytic activity of KPC-1, this is based on a lack of rescue using a catalytically dead form of KPC-1, whereas the wildtype rescue itself is partial (Figure 6B).

1. Experiments which utilized variable amounts of ectopic MNR-1 expression tested only 1 ng/µl (rescue of a null mnr-1(dz175) background) and 20 ng/µl (overexpression phenotype which is kpc-1 null-like), however did not display results using intermediate concentrations (such as the 5 ng/µl or 10 ng/µl, as in (Salzberg et al., 2013, 2014), nor results co-adding KPC-1 to the over-expression injection mix to assay the inter-dependence of both proteins (for example, an injection mix containing 20 ng/µl mnr-1p::mnr-1 and 20 ng/µl kpc-1p::kpc-1).

2. The dual-labeled MNR-1 seems like a powerful tool to track protein localization and intensity using timelapse imaging, in particular of any shedding from the hypodermis, for instance in vesicles, and whether the protein appears in complete or cleaved form. Uncleaved protein is expected to be red and green while cleaved ectodomain will be red only and intracellular and TM protein will be green only. Extracellular vesicles can be derived from the hypodermis and then fuse to the PVD and coelomocytes. Time lapse microscopy using RFP-MNR-1-NeonGreen can help distinguish the different scenarios (see Fig. 7).

3. The effect of kpc-1 absence on the shedding of MNR-1 is quantified based on relative fluorescence of extrachromosomal expression of the dual-label MNR-1 to ectopically-expressed tagBFP in coelomocytes, and is missing temporal information as well as additional backgrounds. Is the decrease in red coelomocyte signal intensity of kpc-1 mutants rescued by ser-2p3::kpc-1 expression? Does it remain the same if a similar rescue is performed non-cell-autonomously (e.g. in FLP, as in figure S4, or from the hypodermis itself)? The presence of a green PVD signal in figure 7D,E is not explained – is this bleed-through from the blue channel, or rather true MNR-1 mNeonGreen signal?

© 2023. Published by The Company of Biologists under the terms of the Creative Commons Attribution License (https://creativecommons.org/licenses/by/4.0/).

4. Given that no cleavage is detected when pulling down MNR-1::HA, it may be clearer to repeat the biochemistry using the dual-labeled RFP-MNR-1-mNeonGreen with antibodies against RFP and mNeonGreen, to detect potentially cleaved products which are missing either fragment.

Minor comments:
Additional experiments which may provide further support for the authors' conclusions:
1. Single-copy insertions and temperature shifts using the mnr-1(dz213ts) may also provide valuable ways to assay dosage-dependence and inter-dependence of KPC-1 and MNR-1.

2. Previous reports by (Salzberg et al., 2014) demonstrated reduced growth speed in kpc-1 (gk8) mutants. Does overexpression of MNR-1 also reduce branch growth speeds? Assuming the effect is mediated through mnr-1, the reduced dynamics should be present in the presence of functional MNR-1 (mnr-1(dz213)) at the permissive temperature) and normal when mnr-1 is no longer present (e.g. using mnr-1(dz213)) at the restrictive 25°C temperature).

3. The worms for figure 7 were raised following egg prep (likely hypochlorite treatment, although not mentioned) and starvation synchronization – would autofluorescence be reduced, and the signal clearer, if the synchronization is performed, for instance, by timed egglaying? Is it possible to normalize RFP/mNeonGreen and visualize relative loss of RFP which would indicate cleavage?

4. Is it possible to retain the adhesion complex by ectopic expression of the inversed, DMA-1 from the hypodermis, and MNR-1 from the PVD?

5. Consider performing the complementation studies on a clean background instead of the kpc-1(gk333538)/kpc-1(gk8) background displayed in figure 2E,G.

The paper contains several instances of "data not shown" (recessive nature of the mnr-1 dz213 allele, rescue using an epidermis-driven MNR-1 L135F temperature sensitivity of the mnr-1(dz213) allele). Consider adding the relevant data in some form.

Additionally, there are typos and some missing information in the manuscript which is divided below to issues relating to the main text, the figures, and lastly Material & Methods + Supplemental-related.

Related to the text:
The use of kpc-1(R265Q) in the text and the allele kpc-1(gk333538) in the figures is confusing – perhaps it will be clearer to present it as R265Q in the figures as well, and add the allele in the figure legend. Similarly, mnr-1 (dz213) and L135F are used interchangeably.

Consider revising descriptions of "perfectly conserved residues" if referring to only a handful of examples, unless an extensive comparison was carried out and is otherwise not mentioned.

A 'partial' rescue and a 'full' rescue are not described in terms of the assayed phenotype or how it was scored, and no representative images are provided. Is the partial phenotype assayed by distribution along the PVD? Percentage of disorganized candelabras?

In the abstract, the sentence "we find increased amounts of MNR-1/Menorin, both overall and in a set of scavenger cells" is not clear – increased where?

Compared with what?

The authors state "Some MNR-1/Menorin function is required for efficient suppression of partial loss of kpc-1/Furin function because (1) the suppression is dosage sensitive (Fig.2G) and (2) the mnr-1(dz175) putative null allele could not suppress the kpc-1(R265Q) hypomorphic phenotype (Fig.S2C). Fourth, over expression of the MNR-1/Menorin L135F mutant cDNA driven by an epidermal promoter completely rescued the mnr-1/MNR-1 null phenotype in kpc-1(R265Q); mnr-1(dz175) double mutant animals (data not shown)." Replace 'Fourth' with (3).

© 2023. Published by The Company of Biologists under the terms of the Creative Commons Attribution License (https://creativecommons.org/licenses/by/4.0/).

Consider rephrasing “individual dendritic trees of the arbor” in the introduction
In the discussion, the authors mention “epidermal SAX-1/L1CAM”, possibly referring to SAX-7?
Related to the figures:
In all panels, y-axis tick legends are absent except for the maximal value.
While it is possible to infer each tick mark value, this is cumbersome, and will be clearer if all values will be shown. This is particularly true for ratio measurements, where the baseline ‘1’ needs to be clear (e.g. in figure 3E,F).
Figure 1C lists an allele which was lost before it was characterized, and as such may be uninformative to include.

Figure 2F and G seem to contain the same datasets used for control, kpc-1(gk333538), and kpc-1(gk333538);mnr-1(dz213).

Figure 2 panel G second genotype from the left reads mnr-1(dz13), presumably this is (dz213)

Figure 3 panel C shows a hypomorphic phenotype if the first two rows are indeed sibling lines of 20 ng/µl injection mixes for MNR-1, with one showing a strong gradient to the phenotype and the other is evenly distributed. This seems to be the case for the bottom alleles of kpc-1 as well. Are they indeed the null and the weak loss-of-function phenotypes? This should be more clearly explained.

In figure 4 panels C,D,E, the separation of panels into green and red channels is confusing in light of the legend description of wyIs581 red PVD marker used as the red channel does not appear to be a membrane-bound uniform highly-expressed PVD marker. The background dark green tracing/ signal needs to be explained, for example if green and red channels represent individual sliced of different Z heights, while the background is a tracing of the red channel PVD projection. The same holds true for Supplementary figure S3. The underlying PVD marker (presumably F49H12.4p::tagBFP) is not mentioned in the panels of legend.

Similarly, the background rol-6 mutation is not mentioned (although this is specified in the methods). It is unclear which construct was used from the listed dzEx1881 and dzEx1894 – one is a dpy-7p-driven mCherry tagged mnr-1 while the other is formally listed as dpy-1p and tagRFP. Were both strains utilized?

Figure 4 panels C-E have arrowheads which are not specified in the legend.

Figure 4C. Where is the quantification of these experiments?

Figure 5 Are the arrowheads pointing to intracellular vesicles. Have the authors observed in time lapse to determine if they are vesicular carriers instead of plasma membrane localization?

Figure 7B non-specific band is not marked or explained. The red and white open arrowheads are not explained in the legend.

As mentioned above, figure 7D,E shows the PVD in the green channel, however it I not explained whether this is bleed through from the blue channel or true green signal?

If the authors can perform the IP (Fig 7B) using the RFP-MNR-1-NeonGreen and using antibodies against the tags they will be able to determine if there is cleavage dependent on KPC-1 activity.

Fig. 7D and E. Can the authors rule out a scenario in which MNR-1 gets to the PVD from the skin in extracellular vesicles that merge with the PVD and then MNR-1 is cleaved in the PVD by KPC-1?

Figure 7 legend F refers to an unspecified figure SX?

Related to Materials and Methods and Supplemental material:

Some extrachromosomal arrays are not described or referenced to in the text or supplementary information. These include: wdIs52, wdIs53; wyIs581; hmIs4. Note that while dzIs49 and dzIs53 are explained in the material and methods, they appear at times in the figures without explicit

© 2023. Published by The Company of Biologists under the terms of the Creative Commons Attribution License (https://creativecommons.org/licenses/by/4.0/).

description, and are also not expanded in the supplementary table. Also, while dzIs49 is referenced, dzIs53 is not cited.

Although not required, figures S1A and B would benefit from the colors being consistent, namely – a similar color for a WT-like phenotype in both panels.

The asterisks in table S1C are not explained. If they describe the nature/severity of the hypomorphic phenotype? And if it represents statistical significance, how is that calculated?

In figure S2B, the definition of 'Full rescue' and 'Partial Rescue' of the phenotype (assuming it is the kpc-1 null phenotype) is not described and would be helpful given the variability of the kpc-1 loss-of-function phenotype in different alleles (even the characterization involved a qualitative assessment of loss-of-self avoidance, of tertiary branch formation etc.)

Figure S2H and its description lists 1° when referring to second-order branches.

Figure S3B-D Are the arrowheads pointing to vesicles that move or to high levels at the plasma membrane?

Figure S5 legend is missing panel C.

Figure S5B marks KPC-1(H262A) which should be KPC-1(H262A)::sfGFP.

There is a him-5 mutation in the background of some of the strains, and although these strains seem to be used in the context of transgenic / non-transgenic sibling on an extrachromosomal injection, can the authors clarify the use of this background and its phenotypes?

Page 15 "to processes"

Figure S6 Why did the authors use I-TASSER instead of AlphaFold2?

References:
Salzberg, Yehuda, et al. "Skin-derived cues control arborization of sensory dendrites in Caenorhabditis elegans." Cell 155.2 (2013): 308-320.

Salzberg, Yehuda, Nelson J. Ramirez-Suarez, and Hannes E. Bülow. "The proprotein convertase KPC-1/furin controls branching and self-avoidance of sensory dendrites in Caenorhabditis elegans." PLoS genetics 10.9 (2014): e1004657.

First revision

Author response to reviewers' comments

As you will see, the referees express considerable interest in your work, but have some significant criticisms and suggestions for improving your manuscript. If you are able to revise the manuscript along the lines suggested, I will be happy to receive a revised version of the manuscript. Please also note that Development will normally permit only one round of major revision. If it would be helpful, you are welcome to contact us to discuss your revision in greater detail. Please send us a point-by-point response indicating your plans for addressing the referee's comments, and we will look over this and provide further guidance.

Reviewer 1 Advance Summary and Potential Significance to Field...
In this paper, Ramirez-Suarez, Buelow and colleagues explore how balance between the proprotein convertase KPC-1/Furin and the adhesion molecule MNR-1/Menorin regulates dendrite arborization of the multidendritic PVD neurons in C. elegans. The highly structured PVD dendrite morphology makes it a perfect system to examine molecular signals that instruct such an elaborate neuronal architecture. It has long been a puzzle how a proprotein convertase regulates dendrite

© 2023. Published by The Company of Biologists under the terms of the Creative Commons Attribution License (https://creativecommons.org/licenses/by/4.0/).

morphologies, as it is widely assumed that substrates of this proprotein convertase are within the dendrites/neurons. Now, in the current study, the authors entertain an intriguing idea that KPC-1 regulates an adhesive ligand in trans in the substratum over which the dendrites grow. They leverage the power of nematode genetics to reveal that the PVD phenotypes of a kpc-1 hypomorphic mutant, previously thought to be due to increased DMA-1 receptor levels on the dendrite membrane, could be ameliorated by decreasing MNR-1 levels. They went on to address whether KPC-1 can function as a secreted protease, and whether it regulates the levels of putative adhesive ligands, in particular MNR-1. Most of the genetic experiments were well designed and rigorously executed, and the interpretation of the data was mostly clear and convincing. However, experimental evidence supporting that KPC-1 regulates MNR-1 level in a cell-non-autonomous fashion is controversial (see below). The following issues need to be addressed by additional experiments or revision of the manuscript, before the paper could be considered for publication at Development.

We thank the reviewer for the generally positive comments and suggestions, which we address below.

Reviewer 1 Comments for the Author...
Major points:
1. The authors should elaborate more in the Introduction how the distribution of MNR-1/SAX-7 ligand complex in the hypodermis shapes the stereotyped dendritic arborization of PVD. In particular, the tertiary dendrites of PVD grow along the sublateral boundaries of the body wall muscles. This spatial relationship between the PVD dendrites and the neighboring muscles or hypodermal cells needs to be explained in more details, so that the readers can understand experiments in Fig. 4C-4E. They are also advised to introduce the spectrum of phenotypes seen in kpc-1 and mnr-1 mutant alleles of various strength, from self-avoidance defects to stalled branch growth or even a complete collapse of the dendritic arborization.

Thank you for this suggestion. We now include more detail in the introduction (l. 60-72) to make the paper more accessible to general readers. We have also amended the schematic in Figure 1A and the legend to illustrate the lateral nerve tract and the tertiary line. Finally, we also better explain the range of defects that are observed in PVD patterning in different *kpc-1* alleles (l.152-153.

2. The most significant problem of this paper lies in Fig. 7, where the authors claim that KPC-1 regulates the overall levels of MNR-1. If KPC-1 cleaves MNR-1 at aa486 to produce a 55.6 kDa fragment, one would predict that this fragment will decrease in the kpc-1 null mutant. The increased level of MNR-1 in the kpc-1 mutant is opposite to this prediction, although it is consistent with mnr-1 mutation being epistatic to the kpc-1 mutation (Fig. S2C). To settle this issue, one may need to mutate the putative KPC-1 sites of MNR-1, but this is not trivial. In Fig. S7C, the level of LECT-2 seems to be significantly decreased in the kpc-1 mutant. An LECT-2/tubulin ratio should be provided before one concludes whether the level of LECT-2 is affected in the kpc-1 mutant. In Fig. 7D and 7E, it seems that signals of tagRFP and NeonGreen were significantly dimmer in the kpc-1 mutant, compared to those in the wild type. This could affect the signal intensity of tagRFP in the coelomocytes - this is not a problem of the coelomocytes in the kpc-1 mutant, but rather the possibility that kpc-1 regulates mnr-1 expression should be excluded. As the authors claim that KPC-1 acts in trans to regulate MNR-1 levels in a different tissue, these issues should be rigorously addressed. Depending on the results of additional experiments, “in-trans” in the title of the paper may need to be tuned down.

Thank you for these suggestions. We have made a substantial experimental effort to address these issues. In the initially presented experiments, we had used immunoprecipitations of a strain in which MNR-1 was endogenously tagged with HA to get a sense of the amount of MNR-1. Since immunoprecipitations are at best semi-quantitative and do not allow to relate the amounts to tubulin, we have now optimized lysis and Western Blots to a degree that we were able to directly detect MNR::HA without prior precipitation. These much cleaner biochemical experiments show unambiguously, and repeatedly that the total amount of MNR-1::HA is increased in *kpc-1* mutants using tubulin as a benchmark (see revised Fig.7). In a similar vein and as requested by this reviewer, we have quantified and related the amounts of LECT-2/Chondromodulin II to tubulin. These measurements of endogenous protein LECT-2 protein levels reveal that the total amount of

© 2023. Published by The Company of Biologists under the terms of the Creative Commons Attribution License (https://creativecommons.org/licenses/by/4.0/).

LECT-2 is reduced in *kpc-1* mutant animals (see new Fig.7). We have also performed experiments suggested by reviewer 2 using the N-terminally and C-terminally tagged transgenes (see also response to reviewer 2 for details). We have repeated these experiments between 5 and 6 times with several permutations by precipitating with an antibody against the N-terminus and found no convincing evidence for cleavage of MNR-1, at least not a detectable amount. The experiments precipitating with an antibody against the C-terminus of MNR-1 were complicated by the fact that both the precipitating antibody and the detecting antibody were mouse antibodies. Regardless, none of these experiments indicated a detectable amount of cleavage. It is of course logically impossible to prove the absence of something (in this case cleavage). Since these experiments were performed with transgenes under control of the rather strong dpy-7 promoter, it is possible that the amounts of transgenic tagRFP::MNR-1::mNG are so large that cleavage by KPC-1 in small amounts is not visible. Alternatively, no direct cleavage may occur, a possibility we now discuss. Lastly, the reviewer was concerned with the images of the transgenic strain carrying the dually labeled MNR-1 transgene. We have repeated all the experiments with an strain, in which the transgene carrying the N-terminally and C-terminally tagged MNR-1 was integrated, and quantified fluorescence in both wild type and *kpc-1* mutant animals in both the coelomocytes and the epidermis. Again, we find that the amount of fluorophore (red) that marks the N-terminus of *mnr-1* is reduced in *kpc-8* mutants in coelomocytes whereas the C-terminus in the epidermis remains unchanged. In addition, as a result of a suggestion by reviewer 2, we determined colocalization of red and green signal in the epidermis and found near perfect correlation between both signals. This data is now also included in a revised figure S7G. Taken together, it appears as if we cannot conclusively address whether MNR-1 is directly or indirectly cleaved/shed by KPC-1. A definitive answer to the question will likely require an different, endogenously tagged MNR-1. While insertion of the HA-tag in *mnr-1* does not compromise *mnr-1* function, insertion of larger fragments and fluorophores impedes *mnr-1* function our unpublished observations). In other words, despite several attempts, we have not been able to visualize endogenous MNR-1 protein. While we are continuing our efforts, we do not have a conclusive answer at this time. Irrespective of these challenges, the central conclusion of our experiments, both based on genetic and biochemical evidence, unequivocally shows that (1) Menorin is required for the phenotype observed in *kpc-1* mutants, that (2) genetically *kpc-1* is a negative regulator of *mnr-1* and that (3) MNR-1 protein levels are negatively regulated by *kpc-1*. We have toned down the title and removed the reference to 'in trans' in the title and in the manuscript but believe that the title otherwise accurately reflects our findings.

3. Fig. 2E-2G: There are a couple of problems here. (1) Please explain the rationale of analyzing kpc-1(gk333538)/kpc-1(gk8); mnr-1(dz213)/mnr-1(dz175). (2) Data of the control, kpc-1(gk333538) and kpc-1(gk333538); mnr-1(dz213) in 2F seem to be re-used in Fig. 2G. If so, Fig. 2F should be removed, as Fig. 2G contains all the data. (3) In Fig. 2G, mnr-1(dz13) should be mnr-1(dz213).

We now explain the rationale for our experiment of putting both hypomorphic alleles simultaneously over a null allele, namely that even as single copies, reducing *mnr-1* function can suppress loss of *kpc-1* function. We also use this data to show that dz213 fails to complement the dz175 *mnr-1* null allele suggesting that *dz213* is a loss of functional allele. Further, as suggested by the reviewer, we have removed Figure 2F to reduce redundancy of data presentation. We have also corrected the typo in original Figure 2G (now Figure 2F).

4. Fig. 3: In 3E, it would be nice if the PVD defects of the kpc-1(gk333538) could be directly compared with those of low-dose MNR-1 overexpression in the wild-type background. In 3C, an image of kpc-1(gk8) was shown. However, in 3F, the data of kpc-1(dz254) were presented. The reason for this is apparently that kpc-1(gk8) is so severe that characterization of self-avoidance is basically impossible. If so, the authors should explain this in more details, and make it consistent to present the same kpc-1 mutant for the image and quantification.

We thank the reviewer for these suggestions. We agree that the proposed comparison may be nice, but we believe that we already make a similar comparison in Fig.3F, i.e. the comparison between the dz254 hypomorphic allele and the low expressing mnr-1 (in a mnr-1 mutant background, which results in rescue) and a high expressing mnr-1 (in a mnr-1 mutant, which leads to a kpc-2 hypomorphic phenotype). In Figure 3C we have added a panel for kpc-1(dz254) to allow for easier comparison of the data in Figure 3F. Lastly, we now include a sentence to read "Note that when *kpc-1* function is completely lost (*kpc-1(gk8)*) dendrites become essentially trapped in the vicinity

© 2023. Published by The Company of Biologists under the terms of the Creative Commons Attribution License (https://creativecommons.org/licenses/by/4.0/).

of the primary dendrite and self avoidance defects can not be detected." on p. 9, l183 This should clarify why self-avoidance in the *kpc-1* null mutant can not be determined.

5. Fig. 4C-4E: The layout of these panels/images should be significantly modified. The pseudocolor coding is a bit confusing, especially in 4D and 4E. The brightness and contrast of these images need to be improved. Fig. 5A looks much better. (1) For all monochromic channels (green/red), please annotate them as "ser2p3::DMA-1::GFP" and "ser2p3::myr::mCherry", respectively. (2) Please add dual-color merge images for DMA-1/PVD that allow the readers to see DMA-1 reruitment to the tertiary dendrites in 4C but not in 4D or 4E. (3) The authors should show MNR-1 expression in the muscles (fused to tagBFP, etc). From the current images, it is hard to imagine whether the tertiary dendrites are trapped on the muscle surface, or whether MNR-1 on the muscle membrane is redistributed by binding to DMA-1 on the PVD dendrites. Or at least the muscle borders should be shown by DIC as in Fig. S3B-3D.

We appreciate and concur with the suggestions to improve Figure 4. We have added a number of panels and improved the labeling as well as improved brightness and contrast. Additionally, we have labeled the different channels as requested and added both a dual-color image and DIC images. Lastly, we outlined the muscle cells, which now clearly show that the tertiary dendrites are 'coming to MENORIN' and not 'MENORIN to the tertiary dendrites' along the epidermis.

6. Page 12: "We therefore asked whether … can act locally or at a distance….. substrate can function in a diffusible form or not". I suggest the authors rephrase this statement, as it is both vague and confusing in what the experiment is trying to address. The argument and the experimental design here are also perplexing. The results in Fig. S4 basically show that kpc-1 acts cell-autonomously, which is consistent with the data that a secreted KPC-1 is non-functional. This experiment (FLP/PVD cross-rescue) does not add much to our understanding of the KPC-1 mechanisms. If the authors would like to keep these data, I suggest that they be combined with the cell-autonomous rescue experiments of PVD (Fig. 5) but not as a separate section.

We have reorganized this section and combined it with the section of the cell-autonomous rescue experiments as suggested by the reviewer.

Minor points:
1. Please add line numbers to help the review process go more efficiently.

Line numbers have been added.

2. Abstract: "Lastly, we find increased amounts of MNR-1…." Please clarify under what conditions/genetic backgrounds is increased MNR-1 in the coelomocytes detected.

This has been corrected and a phrase "in *kpc-1/Furin* mutants" has been added.

3. I suggest the authors to replace "semblance" with "similarity" (Page 4, 10).

We have replaced "semblance" with "similarity".

4. In Fig. 4B, please use arrows to highlight the secondary PVD dendrites where levels of DMA-1::GFP were quantified.

Thank you for this clarifying suggestion, which we have implemented by inserting a dashed white box. We also indicate 1° and 3° dendrites for additional clarity.

5. The y-axis labels of Fig. 5C, 5D, 6B, 6D should be "% defective PVD neurons"?

Thank you, this has been corrected.

6. Fig. S4 was cited much later than Fig. S5. The authors may want to re-order the supplemental figures so that they match the order of citation in the paper.

Thank you, the supplementary figures have been reordered according to call out sequence.

© 2023. Published by The Company of Biologists under the terms of the Creative Commons Attribution License (https://creativecommons.org/licenses/by/4.0/).

7. Are KPC-1 and DMA-1 colocalized or in proximity to each other on the tertiary PVD branches? The experiment to test this should be straightforward.

Thank you for this suggestion. It would indeed be interesting to see whether KPC-1 and DMA-1 colocalize in 3° dendrites. Unfortunately, both of our transgenic reporter strains carry green fluorescent proteins, making this experiment difficult with existing reagents. We are currently in the process of systematically labeling the components of the Menorin complex with endogenous reporters and hope to address this question in the future.

8. Fig. S5B: Please show the localization of KPC-1(H262A) in the tertiary PVD dendrites. The current images only show KPC-1(H262A) in the axon and the primary dendrite, but it is the tertiary dendrite where KPC-1 is supposed to act to regulate dendrite morphogenesis.

We thank the reviewer for this suggestion. Unfortunately, we were not able to visualize convincingly the KPC-1(H262A) reporter in higher order dendrites. In fact, visualizing the KPC-1::GFP was challenging in the first place, a notion that others have comment on before (see Dong et al. 2016 eLife (https://doi.org/10.7554/eLife.11008), who comment in the paragraph immediately before the discussion: "We made several attempts to visualize KPC-1 in the PVD neuron. KPC-1 tagged on both its N- and C-termini produced very weak fluorescent signal." We hope that with the advent of more sensitive methods or fluorescent proteins better localization of KPC-1 will be possible.

9. Page 15: “SAX-1/L1CAM” should be “SAX-7/L1CAM”.

Thank you, this has been corrected.

Reviewer 2 Advance Summary and Potential Significance to Field...

The paper by Ramirez-Suarez et al., concerns mechanisms of dendritic patterning, expanding on a previously established adhesion complex which serves to pattern a pair of arborized neurons in the nematode Caenorhabditis elegans. The authors provide genetic evidence using partial loss-of-function alleles for the regulation of an adhesion pairing between the skin expressed MNR-1/Menorin and the growing dendrite expressing DMA-1/LRR-TM on the sensory neuron PVD (with some evidence for a second neuron, FLP). This interaction is regulated by KPC-1/Furin, which is required on the neuron membrane in its catalytically active form. Loss of kpc-1 is correlated with decreased accumulation of, possibly cleaved, MNR-1 in coelomocytes. The authors suggest that cleavage or some other regulation in trans by dendritic-anchored KPC-1 on hypodermal MNR-1 is responsible for anchoring the dendritic DMA-1 to the membrane (bound by MNR-1), or, conversely, its removal from the membrane.

Reviewer 2 Comments for the Author...

The paper is well-written, although it could benefit from a clear model depiction, more careful dose-response analysis of different rescue constructs, and additional overexpression experiments and ectopic-tissue expression experiments. Lastly, time-lapse imaging may provide valuable evidence for protein localization and/or cleavage, in particular if coupled with tissue-specific expression of kpc-1 as well as temperature shifts of the mnr-1 (dz213) temperature-sensitive allele described.

Thank you, we appreciate the generally positive comments of reviewer 2, which we address in detail below.

In order to provide sound evidence for the trans interaction which is inferred by the genetic results, we advise the following experiments:

The authors can use the dual-marked MNR-1 (N-terminus tagRFP, C-terminus mNeonGreen) as a key reagent for showing potential cleavage and altered localization under the various genetic backgrounds. This strain can be used for the following: a) time lapse imaging to follow potential shedding of the extracellular fragment of MNR-1, either in vesicles or in another form; b) repeating

© 2023. Published by The Company of Biologists under the terms of the Creative Commons Attribution License (https://creativecommons.org/licenses/by/4.0/).

the IP experiments in the wildtype and kpc-1 mutant using antibodies against RFP and against mNeonGreen, in order to try and detect the two fragments of the protein; c) combining the timelapse mentioned above under the background of the catalytically-dead KPC-1(H262A), or wild-type KPC-1 expressed from a non-PVD promotor.

We have performed extensive immunoprecipitation experiments as suggested by the reviewer with immune precipitations against the N-terminal and C-terminal tags (6 times against the N-terminus and 3 times against the C-terminus). Unfortunately, these experiments have not been conclusive in the sense that we have not obtained convincing evidence for direct cleavage of MNR-1 by KPC-1. The reasons for this are unclear. It could be that (a) MNR-1 is indeed not cleaved by KPC-1 or (b) that the amount of MNR-1 that is cleaved in this highly overexpressing strain (we are using the very strong dpy-7 promoter as part of a multicopy array) is too small to be detected on western blots. We now discuss these results and mention these caveats on p.14 l.313 following.
Based on another excellent suggestion by the reviewer we have investigated colocalization between the red (N-terminal) and green (C-terminal) MNR-1 fragments. Surprisingly, we find near perfect colocalization between the signals in both a wild type and a *kpc-1* mutant background. We mention this fact on p.14 l309. Taken together, these results are somewhat non-intuitive, but a possible explanation is that the amounts of transgenically produced MNR-1 are simple too large to allow detection of small amounts of cleaved products, or MNR-1 is not a direct target of KPC-1. It therefore seems as if some of these questions cannot be resolved with the available reagents. The solution to these issues is likely an endogenously labeled MNR-1 reporter. We have already made several attempts to create such a reporter but have so far been unable to obtain a genome-engineered strain, which does not compromise *mnr-1* function.

Additional experiments may involve a more precise characterization of the inter-dependence of MNR-1 and KPC-1 amounts by coinjecting kpc-1 and mnr-1 at similar levels to determine whether the amounts may be balanced even in an over-expression like state.

See comments below.

The paper requires rewriting to help readers follow the various alleles and their phenotypes. While a simple model is presented in figure 3G, it remains difficult to follow the relationship between the various alleles presented. Additionally, the text, figures, and figure legends need a careful reading for typos as well as missing and/or duplicated information.

We have streamlined the descriptions of alleles to make them consistent. We have made a concerted effort to remove errors in text, figures and figure legends. See also below.

Detailed suggestions – major comments:
While the genetic evidence presented nicely supports the effect of kpc-1 on the hypodermally-expressed mnr-1, and is pinpointed to depend on the catalytic activity of KPC-1, this is based on a lack of rescue using a catalytically dead form of KPC-1, whereas the wildtype rescue itself is partial (Figure 6B).

Thank you for this comment. Several arguments support the conclusion that catalytic activity of KPC-1 is required for PVD patterning. First, our own work showed that missense mutations affecting highly conserved amino acids in the immediate vicinity of the catalytic triad display strong mutant phenotypes in PVD (Salzberg et al. 2014). Second, Dong et al 2016 showed that catalytic activity is required for processing of the prodomain of KPC-1. Finally, our current data suggest that catalytic activity of KPC-1 is required both in a wild type and a truncated construct (Fig.6B,D). We appreciate that rescue does not appear complete, but there is absolutely no rescue with the mutant constructs suggesting that our conclusion is valid that full functionality requires catalytic activity. Regardless, to address the reviewer's concerns, we now say that our data “suggests” that catalytic activity is required rather than that the data “shows” that catalytic activity is required. (line 243)

1. Experiments which utilized variable amounts of ectopic MNR-1 expression tested only 1 ng/µl (rescue of a null mnr-1(dz175) background) and 20 ng/µl (overexpression phenotype which is kpc-1 null-like), however did not display results using intermediate concentrations (such as the 5 ng/µl or 10 ng/µl, as in (Salzberg et al., 2013, 2014), nor results co-adding KPC-1 to the over-

© 2023. Published by The Company of Biologists under the terms of the Creative Commons Attribution License (https://creativecommons.org/licenses/by/4.0/).

expression injection mix to assay the inter-dependence of both proteins (for example, an injection mix containing 20 ng/µl mnr-1p::mnr-1 and 20 ng/µl kpc-1p::kpc-1).

We appreciate the reviewer's comment of potentially using intermediate concentrations of DNA in the hope of obtaining intermediate phenotypes to demonstrate the inter-dependence. We believe, however, that the experiments we did perform already addressed this question in a manner that is more controllable. Specifically, to 'dial down' kpc-1 function, we used an allelic series of five alleles from a null allele (gk8) to a weak hypomorphic allele (dz254). These experiments convincingly show the dependence on the relative amount of retained kpc-1 function. On the other hand, we have used the temperature sensitive *mnr-1(dz213)* allele to demonstrate that suppression of the defects in *kpc-1* hypomorphic alleles is only possible at the permissive temperature, but not at the non-permissive temperature. We therefore believe that the proposed experiments may not reveal a lot we do not already know based on well controlled manipulation of gene function of both *kpc-1* and *mnr-1*.

2. The dual-labeled MNR-1 seems like a powerful tool to track protein localization and intensity using timelapse imaging, in particular of any shedding from the hypodermis, for instance in vesicles, and whether the protein appears in complete or cleaved form. Uncleaved protein is expected to be red and green while cleaved ectodomain will be red only and intracellular and TM protein will be green only. Extracellular vesicles can be derived from the hypodermis and then fuse to the PVD and coelomocytes. Time lapse microscopy using RFP-MNR-1-NeonGreen can help distinguish the different scenarios (see Fig. 7).

The reviewer proposes a set of interesting experiments. We addressed this experimentally by measuring colocalization between red (N-terminal) and green (C-terminal) fluorescence. We found near perfect colocalization (see new Fig.7G), suggesting that the strain contains little if any visible N-terminal fragment. We do not know whether that is because the fraction of cleaved N-terminal fragment is too small compared to the overall amount of dually labeled MNR-1 in this transgenic strain or whether there is no cleavage. We believe that only a reporter for endogenously labeled MNR-1 will ultimately allow to address this question. Despite several attempts we have not yet been able to obtain such a reagent.

3. The effect of kpc-1 absence on the shedding of MNR-1 is quantified based on relative fluorescence of extrachromosomal expression of the dual-label MNR-1 to ectopically-expressed tagBFP in coelomocytes, and is missing temporal information as well as additional backgrounds. Is the decrease in red coelomocyte signal intensity of kpc-1 mutants rescued by ser-2p3::kpc-1 expression? Does it remain the same if a similar rescue is performed non-cell-autonomously (e.g. in FLP, as in figure S4, or from the hypodermis itself)? The presence of a green PVD signal in figure 7D,E is not explained – is this bleed-through from the blue channel, or rather true MNR-1 mNeonGreen signal?

To further corroborate the findings with the extrachromosomal arrays, we now swap the original data with data from the integrated extrachromosomal array. The experiments provided the same results, i.e. we find a decreased amounts of the red signal (putative extracellular portion) in the coelomocytes and no change in epidermal staining. In addition, we have put significant effort in additional biochemical experiments (see also next point). These experiments unambiguously show that the absolute endogenous amount of MNR-1 is increased. We also thank the reviewer for picking up on the green staining of PVD. The explanation for this staining is rather trivial and comes from the fact that the strains we analyzed also carried the *wdIs52* transgene, which we unfortunately omitted in the figure legend. We have corrected the figure legend (now Fig.S7D).

4. Given that no cleavage is detected when pulling down MNR-1::HA, it may be clearer to repeat the biochemistry using the dual-labeled RFP-MNR-1-mNeonGreen with antibodies against RFP and mNeonGreen, to detect potentially cleaved products which are missing either fragment.

As suggested by the reviewer, we have performed extensive immunoprecipitation experiments with immune precipitations against the N-terminal and C-terminal tags (6 times against the N-terminus and 3 times against the C-terminus). Unfortunately, these experiments have not been conclusive in the sense that we do not see evidence for direct cleavage of MNR-1 by KPC-1. The reasons for this are unclear. It could be that (a) MNR-1 is indeed not cleaved at all by KPC-1 or (b) that the amount

© 2023. Published by The Company of Biologists under the terms of the Creative Commons Attribution License (https://creativecommons.org/licenses/by/4.0/).

of MNR-1 that is cleaved in this overexpressing strain is too small to be detected on western blots. It therefore seems as if this important question cannot be resolved with the available reagents. We now discuss these caveats appropriately on p.14, l313.

Minor comments:

Additional experiments which may provide further support for the authors' conclusions:

1. Single-copy insertions and temperature shifts using the mnr-1(dz213ts) may also provide valuable ways to assay dosage-dependence and inter-dependence of KPC-1 and MNR-1.

We agree that single copy insertion of mnr-1 may further corroborate our findings but believe that the careful genetic analysis using *kpc-1* alleles of different severity as well as the temperature shift experiments using the *mnr-1(dz213)* allele already make a convincing point with regard to the inter-dependence of the *kpc-1* and *mnr-1*.

2. Previous reports by (Salzberg et al., 2014) demonstrated reduced growth speed in kpc-1 (gk8) mutants. Does overexpression of MNR-1 also reduce branch growth speeds? Assuming the effect is mediated through mnr-1, the reduced dynamics should be present in the presence of functional MNR-1 (mnr-1(dz213)) at the permissive temperature) and normal when mnr-1 is no longer present (e.g. using mnr-1(dz213)) at the restrictive 25°C temperature).

We completely agree with the reviewer that testing whether overexpressing mnr-1 could have similar effects on the growth speed of dendrites as kpc-1 loss of function could provide additional supporting evidence about the inter-dependence and similarity in phenotypes. This experiment would require first the creation of new transgenic, *mnr-1(dz213)* overexpressing strains. Assuming that at the non-permissive temperature these overexpressing strains look completely wild type (which may or may not be the case), we could indeed perform the time-lapse experiments suggested by the reviewer. We do, however, believe that the experimental effort for these experiments, which would largely corroborate the findings we have already based on a careful study of morphological phenotypes of different alleles, exceeds the benefits of what the reviewer raises as a "minor point".

3. The worms for figure 7 were raised following egg prep (likely hypochlorite treatment, although not mentioned) and starvation synchronization – would autofluorescence be reduced, and the signal clearer, if the synchronization is performed, for instance, by timed egglaying? Is it possible to normalize RFP/mNeonGreen and visualize relative loss of RFP which would indicate cleavage?

We have not repeated the experiments with timed egg-laying, but we have quantified the amount of colocalized red and green signal (and of course also the red-non-green signal and the green-non-red signal. We do not observe any obvious differences between wild type and *kpc-1* mutant animals.

4. Is it possible to retain the adhesion complex by ectopic expression of the inversed, DMA-1 from the hypodermis, and MNR-1 from the PVD?

That is a very interesting thought, that we have not pursued yet. One argument against this is that DMA-1 may not be correctly localized in the hypodermis since we do not know whether SAX-7 and DMA-1 can be part of the same complex in a cis-configuration rather than a trans-configuration. In addition, we know that *hpo-30* is required for proper DMA-1 localization. Again, we do not know whether *hpo-30* is expressed in the epidermis. Once these questions are resolved the proposed approach to 'reengineer' the system may be a powerful tool to gain additional insights.

5. Consider performing the complementation studies on a clean background, instead of the kpc-1(gk333538)/kpc-1(gk8) background displayed in figure 2E,G. The paper contains several instances of "data not shown" (recessive nature of the mnr-1 dz213 allele, rescue using an epidermis-driven MNR-1 L135F, temperature sensitivity of the mnr-1(dz213) allele). Consider adding the relevant data in some form. Additionally, there are typos and some missing information in the

© 2023. Published by The Company of Biologists under the terms of the Creative Commons Attribution License (https://creativecommons.org/licenses/by/4.0/).

manuscript, which is divided below to issues relating to the main text, the figures, and lastly Material & Methods + Supplemental-related.

Thank you for pointing this out. The purpose of this experiment was not sufficiently clear as also pointed out by reviewer 1. We now make this more clear (p.8). We have added some but not all data. We believe that the paper is genetically already quite comprehensive and believe that adding more data to support peripheral points may be more distracting than helpful for a general reader.

Related to the text:
The use of kpc-1(R265Q) in the text and the allele kpc-1(gk333538) in the figures is confusing – perhaps it will be clearer to present it as R265Q in the figures as well, and add the allele in the figure legend. Similarly, mnr-1 (dz213) and L135F are used interchangeably.

The terminology has been unified as suggested

Consider revising descriptions of “perfectly conserved residues” if referring to only a handful of examples, unless an extensive comparison was carried out and is otherwise not mentioned.

We removed the word “perfectly”.

A ‘partial’ rescue and a ‘full’ rescue are not described in terms of the assayed phenotype or how it was scored, and no representative images are provided. Is the partial phenotype assayed by distribution along the PVD? Percentage of disorganized candelabras?

We added the following descriptors directly to the figure legend of Figure S2B: Full rescue was defined as PVDs with proximal menorahs (100 μm anterior to the cell body) with untangled and orthogonally located secondary, tertiary and at least two quaternary branches. Partial rescue was defined as PVDs with proximal menorahs (100 μm anterior to the cell body) composed of untangled and orthogonally located secondary and tertiary of variable length but with one or less quaternary branches.

In the abstract, the sentence “we find increased amounts of MNR-1/Menorin, both overall and in a set of scavenger cells” is not clear – increased where? Compared with what?

We have changed this sentence to read: “..we find increased amounts of MNR-1/Menorin in *kpc-1/Furin* mutants, both overall and in a set of scavenger cells.”

The authors state “Some MNR-1/Menorin function is required for efficient suppression of partial loss of kpc-1/Furin function because (1) the suppression is dosage sensitive (Fig.2G) and (2) the mnr-1(dz175) putative null allele could not suppress the kpc-1(R265Q) hypomorphic phenotype (Fig.S2C). Fourth, over expression of the MNR-1/Menorin L135F mutant cDNA driven by an epidermal promoter completely rescued the mnr-1/MNR-1 null phenotype in kpc-1(R265Q); mnr-1(dz175) double mutant animals (data not shown).” Replace ‘Fourth’ with (3).

Thank you - this has been corrected.

Consider rephrasing “individual dendritic trees of the arbor” in the introduction

We have rephrased this expression to read “the individual units of the dendritic tree have also been named menorahs”.

In the discussion, the authors mention “epidermal SAX-1/L1CAM”, possibly referring to SAX-7?

Thank you - this has been corrected.

Related to the figures:
In all panels, y-axis tick legends are absent except for the maximal value. While it is possible to infer each tick mark value, this is cumbersome, and will be clearer if all values will be shown. This is particularly true for ratio measurements, where the baseline ‘1’ needs to be clear (e.g. in figure 3E,F).

© 2023. Published by The Company of Biologists under the terms of the Creative Commons Attribution License (https://creativecommons.org/licenses/by/4.0/).

Thank you - this has been corrected.

Figure 1C lists an allele which was lost before it was characterized, and as such may be uninformative to include.

This has been removed.

Figure 2F and G seem to contain the same datasets used for control, kpc-1(gk333538), and kpc-1(gk333538);mnr-1(dz213).

Figure 2F has been removed.

Figure 2 panel G second genotype from the left reads mnr-1(dz13), presumably this is (dz213)

Thank you - this has been corrected.

Figure 3 panel C shows a hypomorphic phenotype if the first two rows are indeed sibling lines of 20 ng/µl injection mixes for MNR-1, with one showing a strong gradient to the phenotype and the other is evenly distributed. This seems to be the case for the bottom alleles of kpc-1 as well. Are they indeed the null and the weak loss-of-function phenotypes? This should be more clearly explained.

Thank you for this suggestion. We now explain this much better in the legend to panel 3C: “Note that in panel C, the mnr-1 overexpressing strains display a range of phenotypes from severe self-avoidance defects to complete entrapment of dendrites along the primary dendrite. Similarly, partial loss of function alleles of kpc-1 show a range of self-avoidance defects whereas the kpc-1(gk8) null allele show the entrapment phenotype”.

In figure 4 panels C,D,E, the separation of panels into green and red channels is confusing in light of the legend description of wyIs581 red PVD marker used, as the red channel does not appear to be a membrane-bound uniform highly-expressed PVD marker. The background dark green tracing/ signal needs to be explained, for example if green and red channels represent individual sliced of different Z heights, while the background is a tracing of the red channel PVD projection. The same holds true for Supplementary figure S3. The underlying PVD marker (presumably F49H12.4p::tagBFP) is not mentioned in the panels of legend. Similarly, the background rol-6 mutation is not mentioned (although this is specified in the methods). It is unclear which construct was used from the listed dzEx1881 and dzEx1894 - one is a dpy-7p-driven mCherry tagged mnr-1, while the other is formally listed as dpy-1p and tagRFP. Were both strains utilized?

Thank you, we have redesigned this figure to address these issues and also corrected clerical errors. In addition, we have expanded to describe now the full genotypes and added DIC images. We have applied the same corrections to figure S3. The dzEx1881 mentioned by the reviewer has been removed because it is no longer part of the manuscript and has been deleted. dzEx1894 is now only used in its integrated version (dzIs125) which is presented in Figure S7.

Figure 4 panels C-E have arrowheads which are not specified in the legend.

Thank you, this figure has been generally revised according to suggestions made by reviewer 1. Explanations for arrowheads have been added.

Figure 4C. Where is the quantification of these experiments?

The quantification for these experiments is in Figure 4F.

Figure 5 Are the arrowheads pointing to intracellular vesicles. Have the authors observed in time lapse to determine if they are vesicular carriers instead of plasma membrane localization?

Thank you for this thoughtful question. The kpc-1::GFP reporter is very weak. In the axon we could see moving puncta, suggesting that at least some of the staining is in vesicular carriers. However, in

© 2023. Published by The Company of Biologists under the terms of the Creative Commons Attribution License (https://creativecommons.org/licenses/by/4.0/).

dendrites, the staining was too weak to differentiate between potential diffuse plasma membrane staining and vesicular staining (like has been done for DMA-1::GFP staining). We therefore only allow ourselves the careful conclusion that KPC-1::GFP is localized to a membrane compartment based on the dependence on the transmembrane domain, without specifying with membrane compartment. Better reporter or different approaches are likely required to address this question.

Figure 7B non-specific band is not marked or explained. The red and white open arrowheads are not explained in the legend. As mentioned above, figure 7D,E shows the PVD in the green channel, however it I not explained whether this is bleed through from the blue channel or true green signal? If the authors can perform the IP (Fig 7B) using the RFP-MNR-1-NeonGreen and using antibodies against the tags they will be able to determine if there is cleavage dependent on KPC-1 activity.

Thank you for these comments. Old Figure 7B is no longer part of the manuscript, because we have replaced these Ips of MNR-1::HA with direct Western Blots that allow quantification in relation to alpha tubulin. The green PVD staining is now explained in the legend to the new figure S7. It is due to the presence of the wdIs52 transgene which unfortunately we had missed to mention.

Fig. 7D and E. Can the authors rule out a scenario in which MNR-1 gets to the PVD from the skin in extracellular vesicles that merge with the PVD and then MNR-1 is cleaved in the PVD by KPC-1?

We may not be able to formally exclude this possibility. We consider this likely, however, based on the observations that the physical source of MRN-1 is important. In our 2013 Cell paper we show that expression of MNR-1 and SAX-7 in cis in the seam cells is sufficient to attract the PVD dendrites.

Figure 7 legend F refers to an unspecified figure SX? Related to Materials and Methods and

Thank you for catching this error. This has been removed.

Supplemental material:
Some extrachromosomal arrays are not described or referenced to in the text or supplementary information. These include: wdIs52, wdIs53; wyIs581; hmIs4. Note that while dzIs49 and dzIs53 are explained in the material and methods, they appear at times in the figures without explicit description, and are also not expanded in the supplementary table. Also, while dzIs49 is referenced, dzIs53 is not cited.

Thank you -these errors/omissions have been corrected/added.

Although not required, figures S1A and B would benefit from the colors being consistent, namely - a similar color for a WT-like phenotype in both panels. The asterisks in table S1C are not explained. If they describe the nature/severity of the hypomorphic phenotype? And if it represents statistical significance, how is that calculated?

We presume the referee is referring to Figures S2A, S2B, and S2C? Thank you for the suggestions regarding S2A and S2B, which we have implemented. We have changed the asterisks to + (plus) signs, which indicate, arbirtrarily, the severity of the *kpc-1* hypomorphic self avoidance phenotype: with + barely noticeably, ++ noticeable, +++ very obvious.

In figure S2B, the definition of ‘Full rescue’ and ‘Partial Rescue’ of the phenotype (assuming it is the kpc-1 null phenotype) is not described and would be helpful given the variability of the kpc-1 loss-of-function phenotype in different alleles (even the characterization involved a qualitative assessment of loss-of-self avoidance, of tertiary branch formation etc.)

We have added and explanation of how ‘partial and full’ rescue was defined in the legend to figure S2B.

Figure S2H and its description lists 1° when referring to second-order branches.

We are actually referring to 1° branches in this particular figure. To make this clear we directly call out to this panel from the corresponding section of the text (where we previously only called out to

© 2023. Published by The Company of Biologists under the terms of the Creative Commons Attribution License (https://creativecommons.org/licenses/by/4.0/).

Fig.S2 in general. Thank you for catching this. We now also modified this sentence to read: "). *kpc-1(dz254)* mutant animals display shorter 1° branches and self-avoidance defects like the stronger hypomorphic alleles {Salzberg, 2014 #24143} but not an increased number of 2° branches, when compared to wild type control animals (Fig.S2F,H)"

Figure S3B-D Are the arrowheads pointing to vesicles that move or to high levels at the plasma membrane?

The arrowheads point to high levels of DMA-1::GFP at the plasma membrane. This is now specified in the figure legend.

Figure S5 legend is missing panel C.

Thank you - this has been corrected. Figure S5 is now the new Figure S4.

Figure S5B marks KPC-1(H262A) which should be KPC-1(H262A)::sfGFP. There is a him-5 mutation in the background of some of the strains, and, although these strains seem to be used in the context of transgenic / non-transgenic sibling on an extrachromosomal injection, can the authors clarify the use of this background and its phenotypes?

Over the years, we have on occasion used strains containing a him-5 allele, usually for practical reasons, but always with isogenic controls. Regardless, we have known for a long time that him-5 has no effect on PVD development. Initially, we thought that him-5 is required for PVD morphogenesis, because the him-5(e1490) allele we had been using displayed PVD patterning defects. When we tested the deletion allele ok1896, we did not see any defects in PVD suggesting that the phenotype in e1490 was caused by a linked mutation. It took some time to figure out that the phenotype was caused by a closely linked nonsense mutation in hpo-30, which we named dz189 (Tang et al. 2019). We do not know whether the original e1490 isolate contained this mutation or whether it arose spontaneously in the e1490 allele we happened to use, but we have been using the him-5(ok1896) allele ever since if applicable. We will reorder the e1490 allele from CGC strain collection to clarify this issue and advise the CGC accordingly if the dz189 mutation is contained in the original isolate. For clarification, we have inserted the following sentence in the legend to new Figure S4C: "Note that this strain also harbors the him-5(ok1896) allele, which has no effect on PVD morphogenesis."

Page 15 "to processes"

Thank you - this has been corrected.

Figure S6 Why did the authors use I-TASSER instead of AlphaFold2?

Thank you for this comment. AlphaFold2 was not available at the time and iTASSER allowed modeling based on an existing structure, including removal of the prodomain etc.

References:
Salzberg, Yehuda, et al. "Skin-derived cues control arborization of sensory dendrites in Caenorhabditis elegans." Cell 155.2 (2013): 308-320.

Salzberg, Yehuda, Nelson J. Ramirez-Suarez, and Hannes E. Bülow. "The proprotein convertase KPC-1/furin controls branching and self-avoidance of sensory dendrites in Caenorhabditis elegans." PLoS genetics 10.9 (2014): e1004657.

Second decision letter

MS ID#: DEVELOP/2022/201208

MS TITLE: Convertase-dependent regulation of a membrane tethered ligand tunes dendrite adhesion

© 2023. Published by The Company of Biologists under the terms of the Creative Commons Attribution License (https://creativecommons.org/licenses/by/4.0/).

AUTHORS: Nelson J Ramirez-Suarez, Helen M Belalcazar, Maisha Rahman, Meera Trivedi, Leo T.H. Tang, and Hannes E Buelow

I have now received all the referees reports on the above manuscript, and have reached a decision. The referees' comments are appended below, or you can access them online: please go to BenchPress and click on the 'Manuscripts with Decisions' queue in the Author Area.

The referees are mostly happy with your revisions and we would like to publish a revised manuscript in Development, provided that the referees' remaining comments can be satisfactorily addressed. Please attend to all of the reviewers' comments in your revised manuscript and detail them in your point-by-point response.

Reviewer 1

Advance summary and potential significance to field

This paper reveals molecular mechanisms that govern the processing and maturation of membrane-tethered proteins that instruct dendrite patterning of a developing sensory neurons. Compared to the knowledge of freely diffusible cues in axon guidance, our knowledge about how membrane-tethered signals instruct neuronal architecture, in particular dendrite morphogenesis, is relatively limited. This paper offers novel findings and insights into this field and advances our understanding of the molecular mechanisms of neuronal morphogenesis.

Comments for the author

The authors did a great job in revising the paper, and the results from new experiments provide strong support for their major arguments. By removing some controversial observations and refining other preliminary experiments, the quality of data is significantly improved. I am enthusiastic about the publication of this paper in Development. There are a few places where figure designation for the supplemental materials seems to be wrong, and the authors need to correct them before the paper is accepted. These are:
Line 215: There is no Fig. S3E. It should be S3C-D.
Line 223: It should be Fig. S4A, not S5A.
Line 224: Fig. S6 should be S5.
Line 231: Fig. S4 should be S6.
Line 253: Fig. S5B should be S4C.
Line 286: I don't think I find Fig. S4B related to the described mCherry control for the coelomocyte assay. The authors should carefully check whether the data had been placed correctly in the manuscript.

Reviewer 2

Advance summary and potential significance to field

In this revised manuscript, Ramirez-Suarez et al. have responded to all the comments made by the reviewers. The manuscript reads well, and the figures are clearer. While the conclusions of the paper indeed would have been stronger with biochemical or visual evidence for MNR-1 processing, we appreciate the effort made by the authors in this direction. We agree that there are caveats to the current system of overexpression, and the text has been toned down accordingly.

The authors clearly put a lot of effort into answering all the comments accordingly and new experiments are clearly explained and described.

© 2023. Published by The Company of Biologists under the terms of the Creative Commons Attribution License (https://creativecommons.org/licenses/by/4.0/).

Comments for the author

Major comments:
1. KPC-1 is required strictly cell-autonomously (line 248), suggesting target proteins are indeed non-diffusible, but how does this align with evidence of LECT-2 decreasing? Line 371 2. The title of "…regulation of a membrane-tethered ligand" is perhaps misleading?
The evidence provided may also support an indirect mechanism, as the authors now clearly point out. As such, the effect is not necessarily on a membrane-tethered protein (given evidence for LECT-2 changes) and may not be a ligand at all (if it is indirect). The authors may want to consider to change the title to a take-home-message that reflects the main genetic findings of the revised Ms.
Minor comments:
Regarding the text:
1. Line 320: do the authors mean "revealed no obvious evidence"?
2. Line 371 – add reference to the relevant figure (7)
3. In Figure 4, it is unclear how the extrachromosomal rol-6 serves as control for an integrated strain?
4. Typo: edition = editing? Line 530 5. Typo: remove "and" line 559
Regarding Figure legends:
6. Typo: In Figure 4 A,B legend, "DMA" appears, do the authors mean DMA-1?
7. Typo: In Figure 4 D. legend, correct "whit" to "white" and remove one "and"
8. Typo: In figure 7 legend, "C" seems to refer to panel "B" (LECT-2) and panel "C" (MNR-1) results are described without a placeholder?
9. In figure 4 D, perhaps it would be clearer to say that the transgene is used to control "for the presence of rol-6 in the integrated strain in C" or similar.
Regarding Materials and Methods:
10. The formulas for calculating FIR are unclear, since they seem to form an ABCD/ABCD with no clear difference between numerator and denominator? Can the authors clarify this?
11. When describing Immunopercipitation and western blots, specifications of the anti-HA used (line 545) seem out of place?
12. In the revised Ms the authors used ten or twenty gravid animals for the Western Blot analysis, which provides the strongest evidence for protein amount differences; is this sufficient to make this claim? In a previous manuscript the authors stopdid show a similar result for MNR-1 protein amounts increasing in kpc-1 mutants, which was obtained by immunoprecipitation of five full plates. Does this mean that ten worms are sufficient by Western Blot to replace immunoprecipitation of five plates? Why did the authors choose to replace this panel and omit those previous results? Lack of normalization method? Is this method truly superior (see comments to reviewer #1 point number 2)? Is it even possible to detect the C termini with both mouse antibodies used for immunoprecipitation and detection? (See again comments to reviewer #1 point 2).

Second revision

Author response to reviewers' comments

Reviewer 1 Advance summary and potential significance to field
This paper reveals molecular mechanisms that govern the processing and maturation of membrane-tethered proteins that instruct dendrite patterning of a developing sensory neurons. Compared to the knowledge of freely diffusible cues in axon guidance, our knowledge about how membrane-tethered signals instruct neuronal architecture, in particular dendrite morphogenesis, is relatively limited. This paper offers novel findings and insights into this field and advances our understanding of the molecular mechanisms of neuronal morphogenesis.

Reviewer 1 Comments for the author
The authors did a great job in revising the paper, and the results from new experiments provide strong support for their major arguments. By removing some controversial observations and refining other preliminary experiments, the quality of data is significantly improved. I am enthusiastic

© 2023. Published by The Company of Biologists under the terms of the Creative Commons Attribution License (https://creativecommons.org/licenses/by/4.0/).

about the publication of this paper in Development. There are a few places where figure designation for the supplemental materials seems to be wrong, and the authors need to correct them before the paper is accepted.

We appreciate the positive feedback of our revised manuscript.

These are:

Line 215: There is no Fig. S3E. It should be S3C-D.

This has been corrected.

Line 223: It should be Fig. S4A, not S5A.

This has been corrected.

Line 224: Fig. S6 should be S5.

This has been corrected.

Line 231: Fig. S4 should be S6.

This has been corrected.

Line 253: Fig. S5B should be S4C.

This has been corrected.

Line 286: I don't think I find Fig. S4B related to the described mCherry control for the coelomocyte assay. The authors should carefully check whether the data had been placed correctly in the manuscript.

Thank you for catching this. The correct reference is Fig.S6C.

Reviewer 2 Advance summary and potential significance to field
In this revised manuscript, Ramirez-Suarez et al. have responded to all the comments made by the reviewers. The manuscript reads well, and the figures are clearer. While the conclusions of the paper indeed would have been stronger with biochemical or visual evidence for MNR-1 processing, we appreciate the effort made by the authors in this direction. We agree that there are caveats to the current system of overexpression, and the text has been toned down accordingly. The authors clearly put a lot of effort into answering all the comments accordingly and new experiments are clearly explained and described.

We are delighted by the appreciative comments and that our efforts are recognized.

Reviewer 2 Comments for the author
Major comments:
1. KPC-1 is required strictly cell-autonomously (line 248), suggesting target proteins are indeed non-diffusible, but how does this align with evidence of LECT-2 decreasing? Line 371

Thank you for this comment. The short answer to this question is that we do not know how this works mechanistically. It seems indisputable that kpc-1 functions cell-autonomously on the one hand and, that the amounts of MNR-1:HA and LECT-2::mNG are changed in kpc-1 mutants on the other hand. We have tried to provide a possible explanation for these observations but also admit that additional studies will be required to answer some of the resulting questions when we write; "Unexpectedly, we also observe a decrease of LECT-2/Chondromodulin II in kpc-1 mutants (Fig.7B). A possible explanation is that an excess of available MNR-1/Menorin obscures putative binding sites for LECT-2 in the SAX-7/L1CAM cell adhesion molecule, thereby resulting in less binding opportunity and consequently degradation of LECT-2. Additional biochemical and genetic experiments will be

© 2023. Published by The Company of Biologists under the terms of the Creative Commons Attribution License (https://creativecommons.org/licenses/by/4.0/).

required to resolve this question. Regardless, catalytic activity of KPC-1/Furin is required to regulate directly or indirectly the localization and the amount of the MNR-1 ligand in trans."

2. The title of "…regulation of a membrane-tethered ligand" is perhaps misleading? The evidence provided may also support an indirect mechanism, as the authors now clearly point out. As such, the effect is not necessarily on a membrane-tethered protein (given evidence for LECT-2 changes) and may not be a ligand at all (if it is indirect). The authors may want to consider to change the title to a take-home-message that reflects the main genetic findings of the revised Ms.

Thank you for this comment. We have changed the title to now read "Convertase-dependent regulation of membrane tethered and secreted ligands tunes dendrite adhesion"

Minor comments:
Regarding the text:
1. Line 320: do the authors mean "revealed no obvious evidence"?

Thank you for catching this. This has been corrected.

2. Line 371 – add reference to the relevant figure (7)

This has been corrected.

3. In Figure 4, it is unclear how the extrachromosomal rol-6 serves as control for an integrated strain?

We have tried to clarify this as suggested by the reviewer in point 9 below.

4. Typo: edition = editing? Line 530

Thank you for catching this. This has been corrected.

5. Typo: remove "and" line 559

Thank you for catching this. This has been corrected.

Regarding Figure legends:

6. Typo: In Figure 4 A,B legend, "DMA" appears, do the authors mean DMA-1?

Thank you for catching this. This has been corrected.

7. Typo: In Figure 4 D. legend, correct "whit" to "white" and remove one "and"

Thank you for catching this. This has been corrected.

8. Typo: In figure 7 legend, "C" seems to refer to panel "B" (LECT-2) and panel "C" (MNR-1) results are described without a placeholder?

This has been corrected.

9. In figure 4 D, perhaps it would be clearer to say that the transgene is used to control "for the presence of rol-6 in the integrated strain in C" or similar.

Thank you for this excellent suggestion, which we have implemented.

Regarding Materials and Methods:
10. The formulas for calculating FIR are unclear, since they seem to form an ABCD/ABCD with no clear difference between numerator and denominator? Can the authors clarify this?

© 2023. Published by The Company of Biologists under the terms of the Creative Commons Attribution License (https://creativecommons.org/licenses/by/4.0/).

Thank, these formulas were originally color code. They have now been indexed instead for clarity.

11. When describing Immunopercipitation and western blots, specifications of the anti-HA used (line 545) seem out of place?

Thank you for catching this! The corresponding experiment was removed in the revised version. This has been corrected.

12. In the revised Ms the authors used ten or twenty gravid animals for the Western Blot analysis, which provides the strongest evidence for protein amount differences; is this sufficient to make this claim? In a previous manuscript the authors stopdid show a similar result for MNR-1 protein amounts increasing in kpc-1 mutants, which was obtained by immunoprecipitation of five full plates. Does this mean that ten worms are sufficient by Western Blot to replace immunoprecipitation of five plates? Why did the authors choose to replace this panel and omit those previous results? Lack of normalization method? Is this method truly superior (see comments to reviewer #1 point number 2)? Is it even possible to detect the C termini with both mouse antibodies used for immunoprecipitation and detection? (See again comments to reviewer #1 point 2).

Thank you for this comment. We did indeed remove the immunoprecipitation experiments for MNR-1::HA. The reason for this was that through optimization we were able to directly probe MNR-1::HA on Western Blot. We believe that direct Western Blotting enables more quantitative evaluations than immunoprecipitations because it allows normalization. With regard the worms used for the Western Blots, the reviewer must have inadvertently misread the Materials and Methods. The Western Blots for SAX-7 and LECT-2 were indeed performed with 10 and 20 gravid adult worms, respectively, but the Western Blots for MNR-1:HA required 10 full plates of worms. The corresponding section in the Materials and Methods section reads “For SAX-7::GFP::FLAG and LECT-2::mNG::FLAG Western Blots, ten and twenty gravid adult animals, respectively, were boiled in loading buffer and loaded directly into the gels. Gradient gels (4-12% GenScript) were used for all experiments. To detect endogenous MNR-1::HA, 10 full plates of C. elegans were washed with HBS (25mM Hepes, pH7.5, 150 mM NaCl) and collected in a 15ml falcon tube.”

Third decision letter

MS ID#: DEVELOP/2022/201208

MS TITLE: Convertase-dependent regulation of membrane tethered and secreted ligands tunes dendrite adhesion

AUTHORS: Nelson J Ramirez-Suarez, Helen M Belalcazar, Maisha Rahman, Meera Trivedi, Leo T.H. Tang, and Hannes E Buelow
ARTICLE TYPE: Research Article

I am happy to tell you that your manuscript has been accepted for publication in Development, pending our standard ethics checks.

© 2023. Published by The Company of Biologists under the terms of the Creative Commons Attribution License (https://creativecommons.org/licenses/by/4.0/).
